# Aroma Clouds of Foods: A Step Forward to Unveil Food Aroma Complexity Using GC × GC

**DOI:** 10.3389/fchem.2022.820749

**Published:** 2022-03-01

**Authors:** Sílvia M. Rocha, Carina Pedrosa Costa, Cátia Martins

**Affiliations:** LAQV-REQUIMTE and Departamento de Química, Universidade de Aveiro, Campus Universitário Santiago, Aveiro, Portugal

**Keywords:** foodstuff, beverages, aroma clouds, volatile organic compounds, SPME, GC × GC, data processing, data analysis

## Abstract

The human senses shape the life in several aspects, namely well-being, socialization, health status, and diet, among others. However, only recently, the understanding of this highly sophisticated sensory neuronal pathway has gained new advances. Also, it is known that each olfactory receptor cell expresses only one type of odorant receptor, and each receptor can detect a limited number of odorant substances. Odorant substances are typically volatile or semi-volatile in nature, exhibit low relative molecular weight, and represent a wide variety of chemical families. These molecules may be released from foods, constituting clouds surrounding them, and are responsible for their aroma properties. A single natural aroma may contain a huge number of volatile components, and some of them are present in trace amounts, which make their study especially difficult. Understanding the components of food aromas has become more important than ever with the transformation of food systems and the increased innovation in the food industry. Two-dimensional gas chromatography and time-of-flight mass spectrometry (GC × GC-ToFMS) seems to be a powerful technique for the analytical coverage of the food aromas. Thus, the main purpose of this review is to critically discuss the potential of the GC × GC–based methodologies, combined with a headspace solvent-free microextraction technique, in tandem with data processing and data analysis, as a useful tool to the analysis of the chemical aroma clouds of foods. Due to the broad and complex nature of the aroma chemistry subject, some concepts and challenges related to the characterization of volatile molecules and the perception of aromas will be presented in advance. All topics covered in this review will be elucidated, as much as possible, with examples reported in recent publications, to make the interpretation of the fascinating world of food aroma chemistry more attractive and perceptive.

## Introduction

Since the beginning of life on earth, smell has been known to allow communication between species, and recover or stimulate memory. The aromas have always fascinated mankind, even in prehistorical times. For instance, the use of scented materials, which were used to flavor various products and environments, mimicking nature’s aromas, is documented since early history. The first odorous materials used were extracts obtained from natural products. The advent of organic chemistry and the advances in analytical instrumentation allowed the isolation and structural identification of odor-active compounds from natural extracts and their consequent syntheses made it possible to produce these compounds on an industrial scale for use in many applications (Pickenhagen, 2017). Nowadays, natural odorants or synthetic ones are largely used in perfumery, agri-food industries, as well as in textile, automobiles, and flavoring industries, among others. Particularly, the food aroma is of major concern for academics or industrials as it represents a significant factor influencing the public’s food-buying decisions and has also been associated with food quality and safety.

**FIGURE 1:**
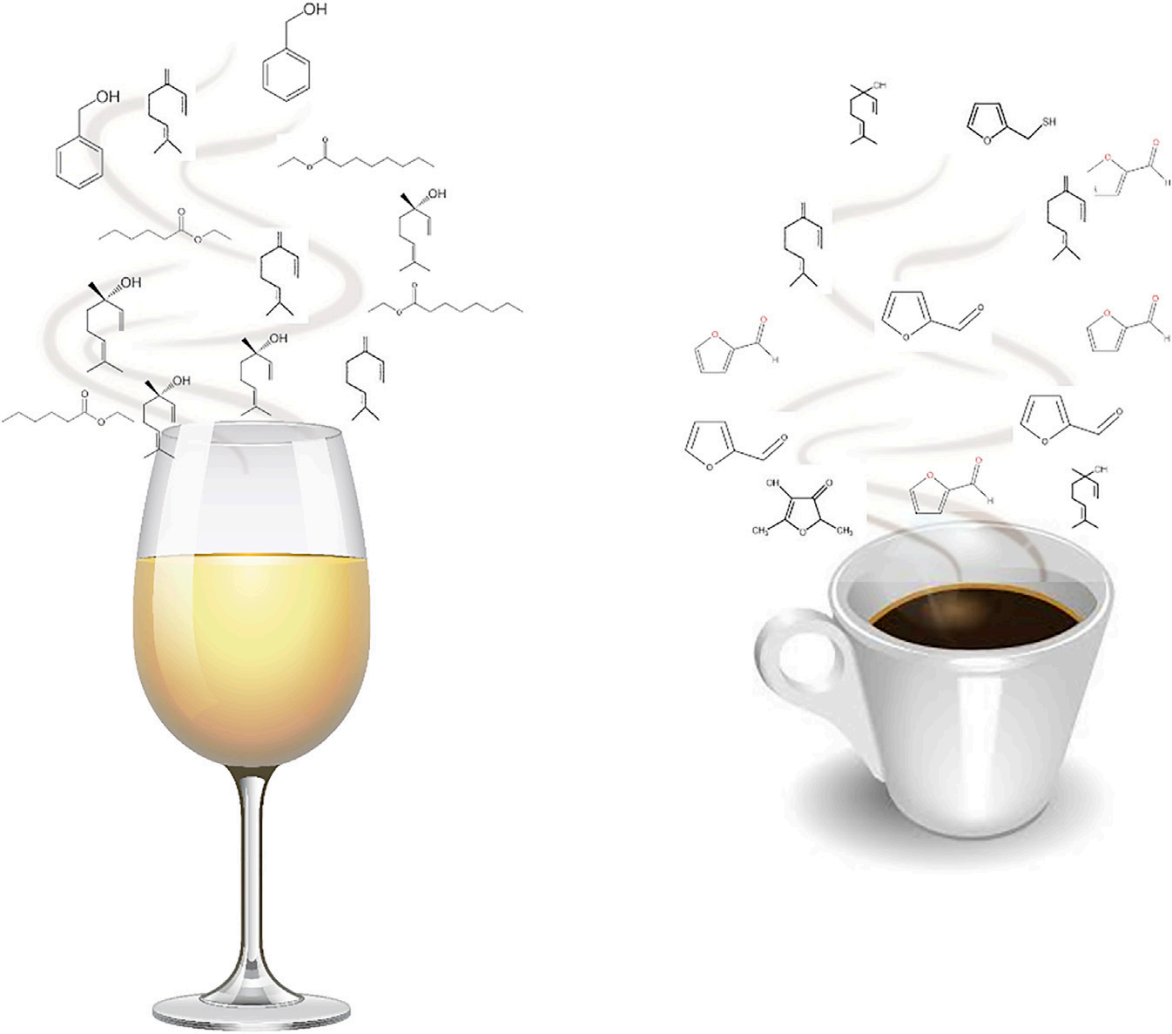
Aroma clouds of foods: a set of odorant volatile and semi-volatile molecules, released from foods, namely from liquid, solid, paste, or others, which are responsible for their aroma properties, here illustrated with white wine and espresso.

Odorous compounds are typically volatile or semi-volatile in nature and have low relative molecular weight (i.e., the majority below 300). Despite this apparently limited range, odorous compounds embody a broad variety of substance classes that comprise diverse structural moieties, such as ester, alcohol, ketone, carboxylic acid, and aldehyde functions, among others; having aromatic or aliphatic forms; and may also include heteroatomic groups (Baldovini, 2017). Odorous molecules may be released from foods, namely from liquid, solid, paste, or others, constituting clouds surrounding them, and are responsible for their aroma notes (Figure 1). The qualitative and quantitative analyses of these molecules, as well as the knowledge of their origin and possible modifications, for instance, during ripening, processing, or storage, are crucial to understand and modulate the aroma properties of foods, and their perception by the consumers.

The aroma of food products is currently evaluated based on sensorial analysis, which translates information closer to what is perceived by humans. However, the implementation of classical sensorial assays is time consuming and expensive, and not all companies and research laboratories are able to maintain the regular functioning of panels. Innovative methodologies that combine sensorial and instrumental data have been developed, namely gas chromatography–olfactometry (GC-O) or electronic noses (Fuller et al., 1964; Delahunty et al., 2006; Giungato et al., 2018; Song and Liu, 2018). Although the chemical characterization by itself fails to reconstruct the final aroma perception that provides useful information about composition and mechanisms of aroma formation, there is nowadays a great interest in understanding the aroma clouds at the level of the molecules that compose them, which justifies the need for the use of robust and highly sensitive techniques. For instance, innovative enhancements, such as smell digitalization and sensomics, which are multi-step analytical approaches, are used to obtain the multipart odor picture of a food, including the identification and accurate quantitation of odorant molecules. Indeed, smell digitalization allows the measuring and chemically revealing of the smells to make them into a digital presentation, which represents cutting-edge research, and usually using artificial intelligence to interpret the odor signatures. Also, sensomics is an approach developed to help in the mapping of both aroma and taste key active molecules, which are perceived by humans’ chemosensory receptors and then integrated by the brain (Schieberle and Hofmann, 2011).

In the last decades, the development of highly sensitive equipment has catalyzed a growing interest in obtaining as detailed information as possible on the volatile composition of foods. These extremely sensitive equipment, as for instance comprehensive two-dimensional gas chromatograph coupled to mass spectrometer with time-of-flight analyzer (GC × GC-ToFMS) or even high-resolution MS, enabled the study of complex samples, with hundreds of components and the measurement of volatile fraction of food products in quantities at the nanogram, picogram, and even lower levels. Also, the solid-phase microextraction (SPME) technique represents a highly efficient sample preparation step before chromatographic determination of volatiles and fulfills the requirements necessary for sustainable development and implementation of green chemistry principles in analytical laboratories (Spietelun et al., 2013; de Souza et al., 2018).

The main purpose of this review is to critically discuss the potential of the GC × GC–based methodologies, combined with a headspace solvent-free microextraction technique (SPME), in tandem with data processing and data analysis, and as a useful tool to the coverage of the chemical aroma clouds of foods. Also because of the comprehensive and complex nature of the aroma chemistry subject, some concepts and challenges related to the characterization of volatile molecules, and the perception of aromas will be presented in advance. All topics covered in this review will be elucidated, as much as possible, with examples reported in recent publications, to make the interpretation of the fascinating world of food aroma chemistry more attractive and perceptive.

### Food Aroma Chemistry: From Olfactation to the Odorant Molecules

Of all five senses, olfaction is the most complex molecular mechanism, as it comprises hundreds of receptor proteins enabling it to detect and discriminate thousands of odorant molecules (Menashe and Lancet, 2006). The understanding of this highly sophisticated sensory neuronal pathway has gained new advances. The sequencing of the human genome and the consequent advances on the associated genomic tools have opened new opportunities to better comprehend this multifaceted biological system.

**FIGURE 2:**
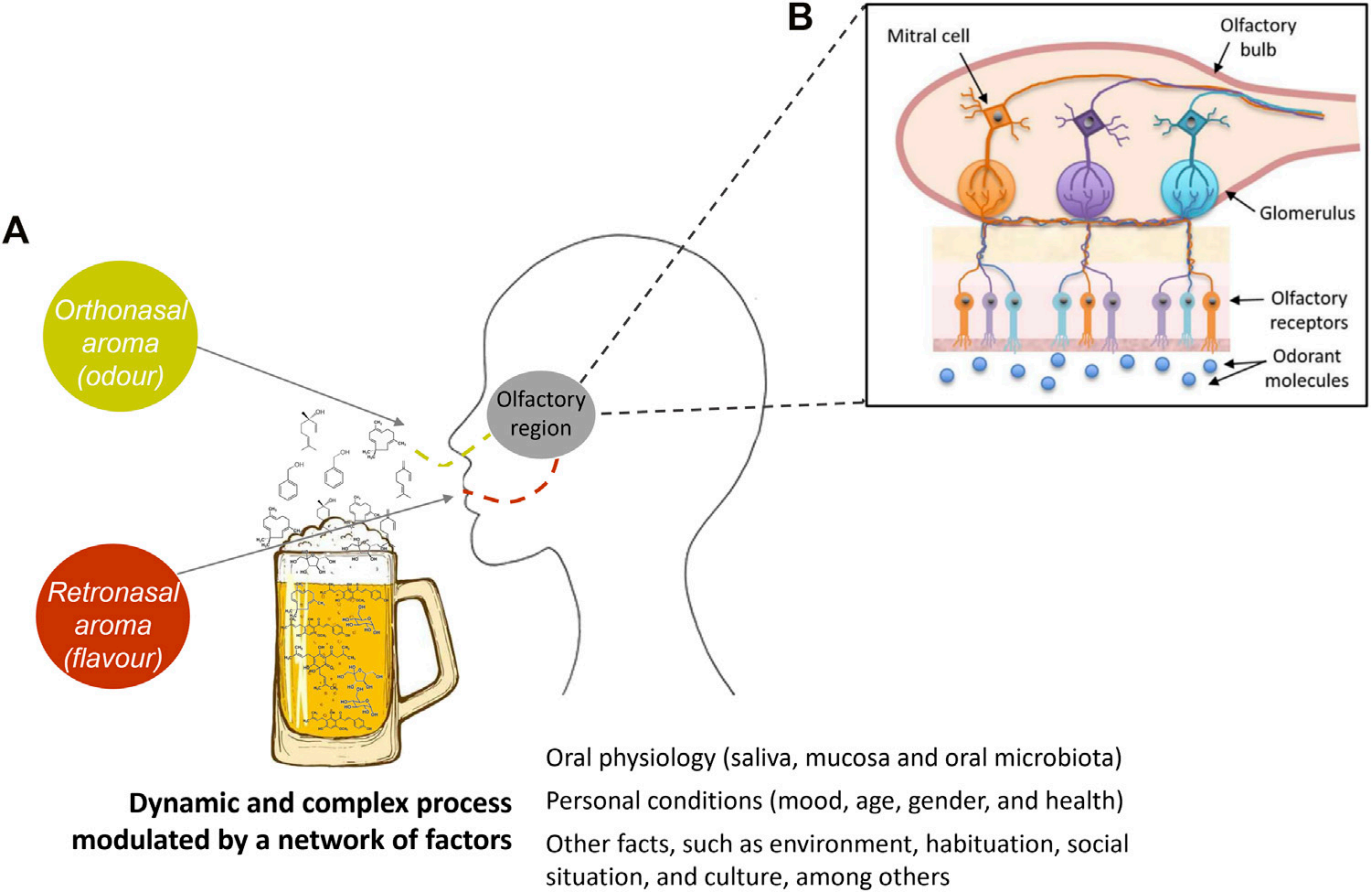
Simplified schematic representation illustrating that odorant molecule release and aroma perception are very complex processes that cover several variables, such as volatile and non-volatile composition of food, and oral physiology-related factors, among others. The odorant substances are perceived by the odor receptor sites of the smell organ. Figure adapted from Kotecha et al. (2018).

According to Buck and Axel (1991), 3% of our genes are used to code the different odorant receptors, which are located on the olfactory receptor cells in the nasal cavity (Figure 2). Each olfactory receptor cell expresses only one type of odorant receptor, and each receptor can detect a limited number of odorant substances. When an odorant receptor is activated by an odorant molecule, an electric signal is produced in the olfactory receptor cell and sent to the brain via nerve processes. Buck and Axel (1991) also showed that each odorant receptor first activates a G protein, to which it is coupled, and which in turn stimulates the formation of cAMP (cyclic adenosine monophosphate). This messenger molecule activates ion channels, which are opened, and the cell is activated. The large family of odorant receptors belongs to the G protein-coupled receptors (GPCR) that differ in certain details, explaining why they are triggered by different odorant molecules (Buck and Axel, 1991; Menashe and Lancet, 2006).

The odorant substances are volatile compounds that are perceived by the odor receptor sites of the smell organ. This perception mechanism can be modulated by several factors associated to the individual, such as physiological characteristics, health condition, previous experiences, culture, and among others (Figure 2). Otherwise, the release of odorant compounds, i.e., their phase transfer from liquid, solid, paste, or other foods into the headspace surrounding, is also a huge element on this dynamic process and depends on numerous intrinsic factors of each molecule, such as interaction, and/or retention phenomena with non-volatile molecules, as well as the environmental conditions (pressure and temperature).

There are two routes of aroma perception: 1) orthonasal detection is when an individual smells food before eating it and the molecules enter the nose through the nostrils and reach the receptors; 2) retronasal detection is when an individual masticates and swallows food during eating (Figure 2). Mastication releases more aroma substances, and these molecules are carried to the throat when the food is swallowed, and then carried to the nose through breath out. Orthonasal or retronasal presentation of odorants evokes different responses, as the way odorant absorption occurs across the mucosa determines the way in which the olfactory epithelium responds (Negoias et al., 2008; Belitz et al., 2009a). The aroma perception, i.e., its recognition, depends on the characteristics of the odorant molecules and their respective concentration that reaches the human olfactory system, and on the nasal and oral physiology mucosa and microbiota. It also depends on the experiences and memories of the individual and their sensory acuity. For example, an individual who has never experienced a certain aroma will never recognize it and associate it with specific notes. Also, for each individual, changes in the thresholds of a given compound and perception of aromas may be observed with moods, time of the biorhythm, and with hunger or satiety, and even with age (Mobley et al., 2013).

More recently, innovative aroma-related initiatives have emerged such as the digital olfaction that mimics the way humans smell. As human sense of smell, digital olfaction mimics the process by which the brain identifies and differentiates odors, by involving a sensor, acting as the nose or receptor for aroma molecules, and associated software that interprets information from the sensor based on a database previously collected and analyzed, and then digitally presents them. It includes an initial advanced chromatographic analysis to chemically characterize the smells. New advances have been performed in this sense by design olfactory metamers—pairs of non-overlapping molecular compositions that generate identical odor percepts (Ravia et al., 2020). With the help of human volunteers (trained and untrained), accurate predictions of perceptual similarity were obtained, suggesting that a valid olfactory measure was achieved, and a step forward to the digitization of smell. The evolution of the digital smell goes toward the digital scent technology (or olfactory technology) that is a technology to sense, transmit, and receive scent-enabled digital media (such as web pages, video games, movies, and music) (Viswanathan and Rajan, 2020).

It is notoriously difficult to study the molecules that produce aromas because a single natural aroma may contain hundreds or even thousands of volatile components, and some of them present in trace amounts. Understanding the components of food aromas has become more important than ever with the transformation of food systems and the increased innovation in the food industry. Indeed, the world has never produced or consumed so much food as in the current decade, which brings enormous challenges and opportunities in several fields. Also, current market trends are changing, namely it has been observed that there is an increasing demand for more natural and less processed products, which may result from the fact that several experts warned that increased consumption of ultra-processed foods has been linked to higher greenhouse gas emissions (da Silva et al., 2021). Otherwise, innovative technological developments in food processing and conservation, as for instance 3D-food printing, allow to obtain foods with novel sensorial characteristics. These products may offer novel consumer sensory experiences and disclose numerous challenges ahead in the aroma food comprehension. Indeed, thousands of foods’ flavor compounds were reported in literature; nevertheless, the typical food aroma and taste is reflected by the combinatorial code of aroma and taste-active key components, considering their specific concentration and biological activity. However, for each food, it is estimated that only 3–40 are true key odorants (Dunkel et al., 2014). It is imperative to understand and deconvolute the puzzle behind the food aroma, at the chemical level, and contribute to comprehend humans’ olfaction sense.

It is common to observe some misconception between the characterization of the food volatile composition and an attempt to understand its aroma, as the study of a food chemistry aroma is much more than the analysis of its volatile composition. As previously reported, of all the food volatile components, only a limited number of them are important for its aroma. An odorous compound, also named aroma contributing substance or aroma-active compound, is primarily present in a higher concentration than its odor threshold (i.e., is the minimum concentration of a substance from which at least 50% of the test subjects can detect and identify a specific odor of a substance–aroma descriptor) (Meilgaard et al., 1999). The aroma potential of each compound may be assessed by calculating its odor active value (OAV) = *c*/*s*, where *c* is the concentration of the compound in a food product and *s* is the olfactory perception threshold (OT) determined in the same type of product. Theoretically, compounds that exhibit OAV >1 were considered to contribute individually to the food aroma and were designated as aroma-active compounds. Furthermore, when the concentration of specific volatile compound is at least 20% of its threshold unit (≥0.2), a sensorial contribution to the food overall aroma should also be considered (Meilgaard et al., 1999; Rocha et al., 2004). In general, the influence of the matrix is low on the aroma descriptors perceived but was high on their intensity (Belitz et al., 2009a). For instance, as illustrated in Table 1, dimethyl sulfide may contribute with a cooked cabbage and sulfury notes, in water or beer, but its threshold value is *ca.* 152 times higher in beer than in water.

**TABLE 1:** Aroma descriptors and odor threshold values of a set of volatile compounds belonging to different chemical families, determined in water, beer, table wine, and lipidic matrices

	Log P^a^	Aroma descriptor	Threshold value (mg/kg)			
			Water	Beer (*ca.* 5–7.5% EtOH)	Table wine (*ca.* 10:90, v/v EtOH/H_2_O)	Lipidic matrices
Butanoic acid	0.8	Cheese, rancid Franco et al. (2004)	0.204 Pino and Mesa (2006)	2.2 Meilgaard (1975)	2.2 Franco et al. (2004)	0.65—olive oil Kalua et al. (2007)
					10 Guth (1997)	50—cream Belitz et al. (2009b)
Hexanoic acid	1.9	Sweaty Uselmann and Schieberle (2015)	3 Pino and Mesa (2006)	8 Meilgaard (1975)	0.42 Ferreira et al. (2000)	0.7—olive oil Kalua et al. (2007)
					3 Guth (1997)	85—cream Belitz et al. (2009b)
Octanoic acid	3.1	Rancid, cheese, fatty Franco et al. (2004)	3 Pino and Mesa (2006)	13 Meilgaard (1975)	10 Franco et al. (2004)	3—olive oil Kalua et al. (2007)
						200—cream Belitz et al. (2009b)
*n*-Butanol	0.9	Medicinal Franco et al. (2004)	0.50 Pino and Mesa (2006)	200 Belitz et al. (2009a)	150 Franco et al. (2004)	—
3-Methylbutanol	1.2	Alcoholic, banana Vanderhaegen et al. (2003a)	0.25 Belitz et al. (2009a)	70 Meilgaard (1975)	30 Guth (1997)	0.1—olive oil Kalua et al. (2007)
			0.30 Pino and Mesa (2006)			
3-Methylbutanal	1.2	Malty Uselmann and Schieberle (2015)	0.00050 Czerny et al. (2008)	0.6 Meilgaard (1975)	—	0.0054—olive oil Kalua et al. (2007)
(*E*)-2-Nonenal	3.6	Papery (cardboard) Vanderhaegen et al. (2003a)	0.00008 Belitz et al. (2009a)	0.00011 Meilgaard (1975)	—	0.9—sunflower oil van Gemert, (2011)
Ethyl hexanoate	2.8	Fruity Uselmann and Schieberle (2015)	0.001 Pino and Mesa (2006)	0.210 Vanderhaegen et al. (2003b)	0.005 Guth (1997)	—
			0.005 Schieberle (1991)			
Furfural	0.4	Sweet, cake, burnt, almond Franco et al. (2004)	3 Pino and Mesa (2006)	—	15 Franco et al. (2004)	—
2,3-Butanedione	−1.3	Butter Uselmann and Schieberle (2015)	0.001 Czerny et al. (2008)	0.15 Meilgaard (1975)	0.1 Franco et al. (2004)	0.003—sunflower oil van Gemert (2011)
						>2–4—butter van Gemert (2011)
γ-Butyrolactone	−0.6	Creamy, oily, fatty, caramel Li et al. (2021b)	20–50 van Gemert (2011)	—	20 Franco et al. (2004)	—
					35 Escudero et al. (2007)	
γ-Hexalactone	0.4	Herbal sweet tobacco peach apricot Li et al. (2021b)	0.26 Boonbumrung et al. (2001)	—	359 Franco et al. (2004)	—
δ-Octalactone	1.5	Sweet, coconut, dairy Li et al. (2021b)	0.200 Czerny et al. (2008)	—	0.386 Ferreira et al. (2000)	2.49—sunflower oil van Gemert (2011)
(E)-β-Damascenone	4.0	Cooked apple-like Uselmann and Schieberle (2015)	0.000004 Schieberle (1991)	0.150 Vanderhaegen et al. (2003b)	0.00005 Guth (1997)	0.011—olive oil Kalua et al. (2007)
			0.000013 Czerny et al. (2008)			
Linalool	3.0	Floral, lemon Thibaud et al. (2020)	0.006 Pino and Mesa (2006)	0.080 Meilgaard (1975)	0.015 Guth (1997)	—
Geraniol	3.6	Floral Thibaud et al. (2020)	0.0011 Czerny et al. (2008)	—	0.020 Escudero et al. (2007)	—
					0.03 Guth (1997)	
α-Terpineol	3.0	Lily, sweet, cake Franco et al. (2004)	1.2 Averbeck and Schieberle (2011)	414 Meilgaard (1975)	5 Franco et al. (2004)	—
Dimethyl sulfide	0.9	Cooked cabbage, sulfury Hui (2010)	0.00033 Belitz et al. (2009a)	0.05 Meilgaard (1975)	0.01 Guth (1997)	—
Guaiacol	1.3	Phenolic Uselmann and Schieberle (2015)	0.00084 Czerny et al. (2008)	0.00388 Sterckx et al. (2011)	0.01 Guth (1997)	0.016—olive oil Kalua et al. (2007)
						0.019–0.050—sunflower oil van Gemert (2011)
4-Vinylguaiacol	1.8	Clove-like Uselmann and Schieberle (2015)	0.003 Pino and Mesa (2006)	0.119 Sterckx et al. (2011)	0.04 Guth (1997)	—
				0.300 Meilgaard (1975)		

a Data obtained from PubChem and FooDB Databases.

Table 1 systematizes the information related with the aroma descriptors and OT values from a set of volatile compounds, belonging to different chemical families, and which were determined in products presenting different physicochemical characteristics. Despite the reported publications, and different panels and methodologies that have been used to determine these sensorial parameters, the comparative analysis of the data from different publications shows that the lower OT values were mainly reported for water. The aroma intensity, expressed here by the OT, depends on several factors associated to the individual and/or panel performance, the physicochemical characteristics of each compound (namely its solubility in water, ethanol/H_2_O or lipidic matrix, and volatility, among others), and on interaction phenomena (synergisms, antagonistic, and/or others) that may occur between the analyte, and other volatile or non-volatile molecules present in the matrix. These last phenomena will be highly marked in alcoholic beverages and lipidic matrices, compared with water. Indeed, higher differences can be observed between OT in the lipidic matrices and the water or ethanol/H_2_O matrices. For instance, the increase of the carbon skeleton in acid components promotes a raise of their log P (parameter to access molecular lipophilicity: butanoic acid—0.8, hexanoic acid—1.9, and octanoic acid—3.1), and compounds with higher log P present higher solubility in the lipidic matrices and consequent lower release to the headspace. This phenomenon may explain the higher OT values of these compounds in lipidic matrices. Butanoic and octanoic acids showed OT values 245 and 67 times higher for cream than the OT reported for water (Table 1). Also, foods’ viscosity has also a significant impact on OT values, as it may modulate the release of volatile compounds from the food product to the surrounding headspace. For example, the sunflower oil and butter present the viscosity at 20°C of 0.063 Pa s (Calligaris et al., 2014) and 427 Pa s (Glibowski et al., 2008), respectively. The lower viscosity from sunflower oil allows a faster gas phase diffusivity of the 2,3-butanedione (log P of −1.3, lipophobic compound) and consequently easier release to the headspace. Thus, 2,3-butanedione requires a lower OT (0.003 mg/kg) to be sensorially perceived in sunflower oil than the OT observed in butter (>2–4 mg/kg) (Table 1).

In fact, foodstuffs and beverages are highly complex matrices that are no longer regarded as a product themselves but as a food system that includes all the activities from the raw material(s) until its consumption. The food composition is the result of a set of factors, such as the raw material(s) composition and their conditions of cultivation, harvesting and storage, as well as the technological processes involved in its production, and subsequent conditions of distribution, storage, and finally the circumstances in which consumption occurs (The Future of Food and Farming: Challenges and choices for global sustainability, 2011; Galanakis, 2020). All these factors play an important role in the food’s chemical composition, nutritional value, and sensory characteristics, including aroma. This holistic view of a food system is critical to comprehend and extract relevant chemical information from the food odorous compounds. On the other hand, information on the food volatile composition can provide key information for the control of raw materials and final products, technological processes, and conditions of transport or storage, among others.

The analytical coverage of the chemical clouds of food needs to be comprehensive and quantitative, and the use of a highly sensitive and high-throughput methodology such as the GC × GC-ToFMS seems to fulfill these requirements.

### The Role of the SPME/GC × GC to Assess Chemical Characterization of Food Aromas

Capture of odorant molecules in a representative way, acquisition of chromatographic data, and its transformation into interpretable and useful information to the chemical characterization of food aromas involve many challenges, intrinsically linked to the peculiarities of volatile and semi-volatile compounds and matrix physicochemical characteristics, as well as of methodologies currently used. Some of these challenges can be listed to exemplify the inherent complexity of aroma chemistry field:

• High diversity of food and beverage products,
• Chemical diversity of volatile and semi-volatile molecules,
• Concentration in a wide dynamic range,
• Multiple sources of variability from sample, analysis methods (extraction and chromatographic ones), workflows, reagents, and so on,
• Lack of analytical standards, particularly due to the presence of unknown molecules,
• The need for robust, reliable data handling and bioinformatics,
• Throughput issues for preparing and analyzing large numbers of samples,
• Lack of information about aroma descriptors and OT values for the volatile and semi-volatile compounds present in a wide range of food products, namely for protected designation of origin products that are highly valued due to their distinctive sensory characteristics,
• Scarcity of information associated with antagonistic or synergistic effects between molecules, and their impact on different food products (matrix effect).

It is also important to point out that the odorant compounds are usually found in very low concentrations, ranging from nanograms to milligrams per liter or kilogram; many of them are highly reactive and thermally labile, and volatilize easily at room temperature (Chen and Ho, 2006; Belitz et al., 2009a). Another issue that also deserves to be highlighted is that, in general, it was observed that there was a higher complexity of the processed foods compared with the fresh ones. Indeed, several dozens or hundreds of volatile and semi-volatile compounds are reported in foodstuffs, as for instance fresh fruits and vegetables, but this number more than doubles when studying the volatile fraction of thermally processed foods, such as coffee, roasted beef, and distillate beverages, among others (Chen and Ho, 2006; Belitz et al., 2009a; Lopes et al., 2021). All these aspects make their analysis a stimulating task, and GC × GC–based methodologies have been considered a promising strategy to overcome these challenges (Amaral and Marriott, 2019; Biedermann and Grob, 2019; Cordero et al., 2019; Rasheed et al., 2021; Welke et al., 2021).

**FIGURE 3:**
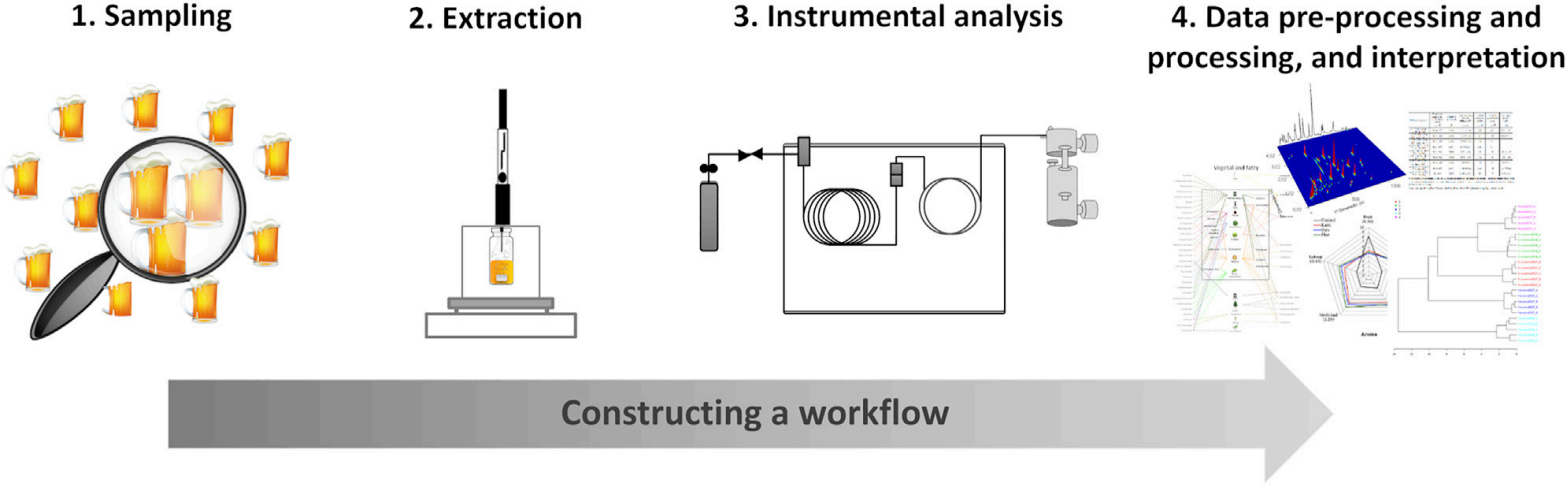
Construction of a workflow toward unveiling the aroma of food items.

As a starting point to the implementation of a methodology, an appropriate workflow should be constructed, as schematically illustrated in Figure 3, which basically consists of four steps: 1) representative sampling; 2) extraction of the volatile and semi-volatile components; 3) data acquisition by chromatography-based methodology; 4) monitoring, pre-processing, and processing of signals, including data interpretation based on the relationship between odorants and sensory properties (Schieberle and Molyneux, 2012).

All the steps of the experiments should be carefully planned, namely the sampling to include sufficient samples representative of the whole population and to assure statistical significance of the results. Attempts to force statistical procedures to fit a data set after sampling can result in conclusions that are not meaningful. Solvent-free microextraction techniques such as SPME, in headspace mode, may represent a powerful tool for extraction of volatiles, including those that compose the aroma clouds (Kataoka et al., 2000; Bressanello et al., 2021). Analysis by GC is a long-established technique, particularly for the study of volatiles and/or aroma constituents (Schieberle and Molyneux, 2012). Advanced gas chromatographs, such as GC × GC, may represent a step further to overcome challenges due the complexity of aroma clouds, being well situated due to its high sensitivity, and chromatographic resolution. Finally, chromatographic data pre-processing and processing, and their combination with sensorial data are crucial to mapping aroma-active compounds, clustering samples, establishing relationship between odorants and food aromas, and explaining formation mechanism of important odorants, among others.

Despite the huge importance of the sampling, in this review the primary attention will be devoted to the extraction, instrumental analysis, and data processing and interpretation steps (data analysis).

### Solid-Phase Microextraction

SPME is a rapid and simple sample preparation technique based on sorption of analytes (absorption and/or adsorption) into a stationary phase, allowing the simultaneous sampling, extraction, and pre-concentration. This technique was developed by Pawliszyn, in the beginning of the 1990 decade (Pawliszyn, 2009), and may be used to extract directly analytes from liquid, solid, and gaseous samples. SPME is a non-exhaustive extraction technique since it only extracts a small analyte fraction, which may be used to characterize the global composition of analytes in the free form (Vas and Vékey, 2004; Pawliszyn, 2009; Souza-Silva et al., 2015; Boyacl et al., 2018). These characteristics allow electing the SPME as a technique especially well positioned to extract the odorant molecules present in the food aroma clouds.

Several other advantages are associated with this technique, namely being a safe and user-friendly procedure, easily automated, and with potential to be miniaturized (Pawliszyn, 2009; Souza-Silva et al., 2015). It can be used routinely in combination with gas chromatography (GC) or liquid chromatography (LC) (Kataoka et al., 2000). Moreover, SPME has the ability to extract analytes at the level of nanograms or even picograms. It is also important to highlight that SPME represents a highly efficient one-step and solvent-free sample preparation technique, having each fiber a high durability, which fulfills the requirements necessary for sustainable development and implementation of green chemistry principles in analytical laboratories (Spietelun et al., 2013; de Souza et al., 2018). Also, considering the current state of SPME development, the price could become an advantage considering the low cost of production. Principally with implementation of this technique in combination with high-throughput equipment, it could become an attractive feature as it can be used multiple times. These attributes are in line with the current concepts about the White Analytical Chemistry that is closer to the idea of sustainable development due to a more holistic view, considering a compromise that avoids an unconditional increase in greenness at the expense of functionality (Nowak et al., 2021).

In fact, SPME fibers can be reused hundreds of times without any compromise to their physicochemical properties and, consequently, extraction efficiency. Nevertheless, it is required to have a regular control of its performance, e.g., regularly a chemical standard with known concentration may be extracted and its concentration may be followed on time. Also, to avoid cross-over contaminations, thermal clean-ups between extractions have to be done. All the previously reported advantages are the justification for the fact that SPME has been successfully used as an extraction technique for a wide range of foodstuffs and beverages (Kataoka et al., 2000; Peñalver et al., 2000; Vesely et al., 2003; Vichi et al., 2003; Pawliszyn, 2009; Giri et al., 2010; Welke et al., 2014; Alves et al., 2015; Santos et al., 2015; Silva et al., 2015; Nunes et al., 2016; Salvador et al., 2016; Xu et al., 2016; Martins et al., 2018, 2020; Fonseca et al., 2020; Bressanello et al., 2021; Lopes et al., 2021; Rocha et al., 2021).

**FIGURE 4:**
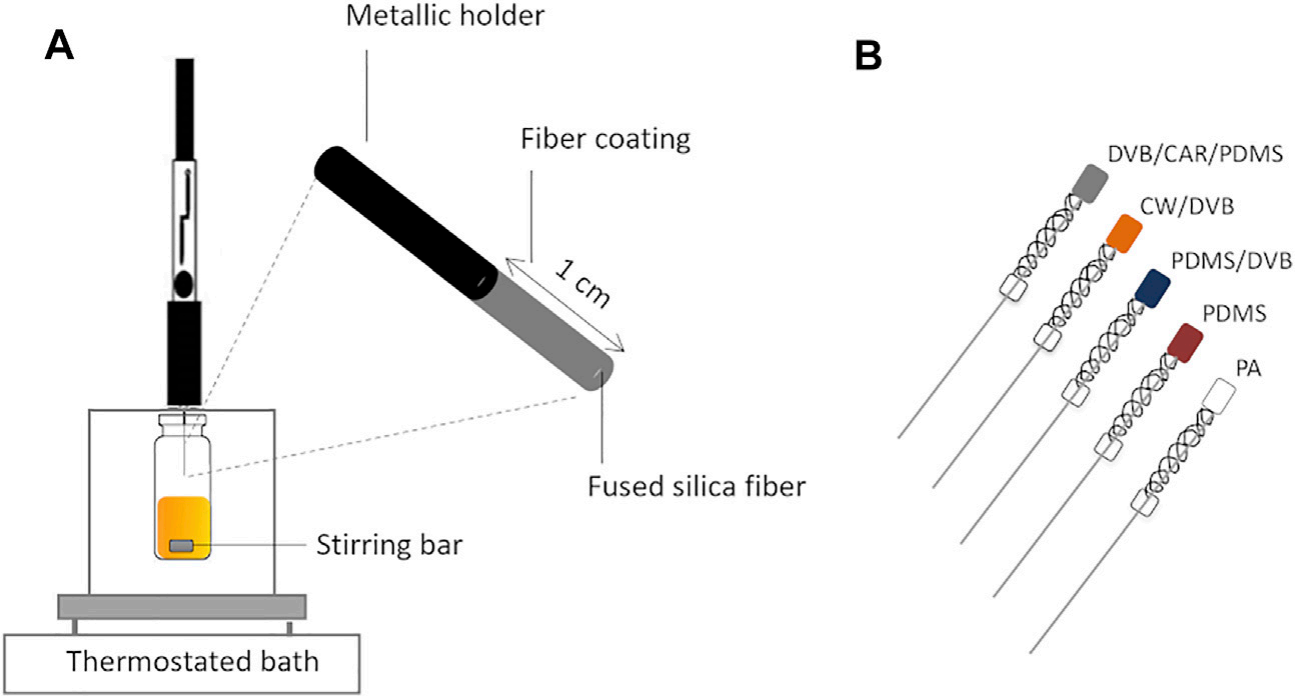
Schematic representation of **(A)** solid-phase microextraction in the headspace mode (HS-SPME) using manual syringe extraction holder and the **(B)** most used commercial stationary phases: DVB/CAR/PDMS—50/30 µm divinylbenzene/carboxen/polydimethylsiloxane; CW/DVB—65 µm carbowax/divinylbenzene; PDMS/DVB—65 µm polydimethylsiloxane/divinylbenzene; PDMS—100 µm polydimethylsiloxane; PA—85 µm polyacrylate.

There are available various SPME geometries, namely fiber, miniaturized fiber (coated-tip), arrow, thin film, in-tube, magnetic nanoparticles, and in-tip (Piri-Moghadam et al., 2017). Nevertheless, fiber, which consists of a needle whose base is usually fused silica coated with a thin layer of a stationary phase inside a syringe, is the most commonly used configuration, and several fiber coatings are commercially available (Figure 4A). Using this configuration, two extraction modes can be performed: immersion or direct extraction (IM-SPME) and headspace extraction (HS-SPME). In the first mode, analytes are released directly from the sample to the fiber coating, once the coated fiber is placed into the sample, especially used for liquid samples. In the headspace extraction mode, the analytes are released from a liquid or solid matrix, being transported through the air barrier, and the vaporized analytes are sorbed by the fiber stationary phase. Thus, no direct contact between fiber and sample occurs, which allows the preservation of the stationary phase against any damage resultant from matrix interferences (Pawliszyn, 2009). The headspace extraction mode is particularly appropriate for capturing volatile and semi-volatile compounds, which are the components of the aroma clouds surrounding foods and beverages (Figure 1).

After fiber exposition, a mass-transfer process takes place, and the analyte’s amount that is sorbed by the stationary phase can be quantified by the following mathematical equation:

$$n=\frac{C_{0}\times V_{f}\times V_{s}\times K}{K\times V_{f}+V_{s}}$$

where *C*_0_ represents the sample’s initial concentration; *V*_f_ and *V*_s_ are the volume of fiber and sample (or headspace), respectively; *K* represents *K*_fh_ × *K*_hs_, in which *K*_fh_ represents the partition coefficient between stationary phase and headspace, and *K*_hs_ is the partition coefficient between the headspace and the sample.

Several experimental parameters determine the extraction efficiency and reproducibility of SPME technique (Rocha et al., 2001; Vas and Vékey, 2004; Pawliszyn, 2009; Souza-Silva et al., 2015), namely:

• SPME fiber coating,
• Extraction temperature,
• Extraction time,
• Salt addition (salting-out effect, applicable specially in the case of aqueous-based samples),
• Stirring effect,
• Sample amount and chemical composition.

Thus, prior to the implementation of a SPME-based methodology, it is crucial to have generic information about the physicochemical characteristics of the sample to be analyzed, and to optimize the previously reported experimental parameters. The matrix effect may have impact on the SPME extraction, i.e., the analytes’ amount sorbed on the SPME fiber is determined by sorption kinetics and also by the analytes’ coefficient distribution between the stationary coating and the food sample, which are intrinsically related with sample physicochemical properties (Rocha et al., 2001). Hence, the various experimental parameters with a relevant impact on the SPME process will be briefly addressed.

Different SPME stationary phases are commercially available, regarding polarity, types of analyte interaction (absorption and/or adsorption), and film thickness and length, although the most common are 1 cm in length (Figure 4B). For the fibers that extract via an adsorptive-type mechanism, such as DVB/CAR/PDMS (divinylbenzene/carboxen/polydimethylsiloxane) and PDMS/DVB (polydimethylsiloxane/divinylbenzene), the analytes interact primarily with the surface of the sorbent coating instead of partitioning into the entire coating and, therefore, the sensitivity of these fibers depends on other factors, such as the surface area and porosity of the material, among others (Shirey, 2012; Godage and Gionfriddo, 2019; Carriço et al., 2020). Lower extraction efficiency of volatile compounds may be observed for PDMS/DVB fiber due to the porosity properties of the DVB that represent some concerns about the analytes’ displacement, and has difficulty to extract analytes with low molecular weight. The DVB/CAR/PDMS fiber, which combines three materials, was developed to overcome the limitations of the CAR/PDMS (carboxen/polydimethylsiloxane) in the desorption of higher molecular weight analytes and PDMS/DVB in difficulty of extracting analytes with low molecular weights. The DVB/CAR/PDMS coating contains both adsorbents that are layered to extend the molecular weight range of analytes extracted with one SPME fiber and the combination with the PDMS, an absorptive-type fiber that also confers a bipolar character (Shirey, 2012; Godage and Gionfriddo, 2019; Carriço et al., 2020). This arrangement explains the high performance of the DVB/CAR/PDMS fiber for a wide range of chemical species and the fact that it is extensively used for profiling of volatile molecules released from various food items, such as table and fortified wine (Perestrelo et al., 2011; Santos et al., 2013; Welke et al., 2014; Whitener et al., 2016; Rocha et al., 2021), beer (Sterckx et al., 2010; Rodriguez-Bencomo et al., 2012; Romero-Medina et al., 2020), sea salt (Donadio et al., 2011; Silva et al., 2015), pear (Wang et al., 2019; Fonseca et al., 2020), distilled beverages (Thibaud et al., 2020), coffee (Ongo et al., 2020; Lopes et al., 2021), elderberry (Salvador et al., 2016), hazelnut (Cordero et al., 2010; Rosso et al., 2020), honey (Čajka et al., 2007; Siegmund et al., 2018), dairy products (Cardinali et al., 2021; Székelyhidi et al., 2021; Liu et al., 2022), virgin olive oil (Purcaro et al., 2014; Ros et al., 2019), and cereals (Buśko et al., 2010). Indeed, DVB/CAR/PDMS fiber exhibited the highest sorption capacity for a wide range of VOCs released from honey, comparing with six other fiber coatings (Čajka et al., 2007). However, not always DVB/CAR/PDMS fiber is the best option to perform SPME in foods, as it was shown for instance in beer analysis (Martins et al., 2015): PDMS/DVB fiber had better reproducibility (expressed as RSD) than DVB/CAR/PDMS fiber (no significant differences in both peak area), using a set of analytes from several chemical families. Furthermore, the representativeness of different volatile extracts from French cider was compared, namely using IM-SPME, HS-SPME, purge and trap, and dynamic headspace (Villière et al., 2012). In this case, HS-SPME using CAR/PDMS as fiber coating was shown to produce the extracts with aroma characteristics, as near as possible, to those of the reference French cider.

Otherwise, diffusion of the analytes through the sorbent coating is a prevailing effect in fibers with absorptive-type mechanism such as PA (polyacrylate) and PDMS (Shirey, 2012) and the more recent fibers based on zwitterionic polymeric ionic liquids (Nan and Anderson, 2018; Pacheco-Fernández et al., 2019; Carriço et al., 2020). Consequently, analytes can freely partition into the sorbent, with little competition among analytes, and the concentration of each analyte at equilibrium is less affected by the presence of other analytes. Despite these fibers that were designed for more specific applications, i.e., for the extraction mainly of polar (PA and zwitterionic polymeric ionic liquids) or non-polar analytes (PDMS), their use can be very useful as they provide representative profiles of the headspace composition.

SPME extraction efficiency also depends on the extraction temperature since it influences the analytes’ vapor pressure and solubility. This parameter interferes in the diffusion coefficients and Henry’s law constants (*K*_H_) of analytes, allowing to modulate the equilibrium time. With increasing extraction temperature, two phenomena can be promoted: the analytes’ volatilization and their consequent transference from liquid or solid phase to the headspace; the analytes’ solubility in the case of liquid matrices (Rocha et al., 2001; Pawliszyn, 2009). Thus, it is crucial to establish an extraction temperature that ensures the best compromise between both phenomena and consequently promote a high extraction efficiency, also ensuring that no sample degradation or artifact formation occurs (Pawliszyn, 2009). Particular care must be taken to prevent the formation of Maillard compounds, which may be produced during the extraction step (Villamiel et al., 2006). For instance, regarding beer volatile profiling, the SPME extraction temperature usually varied between 20 and 60°C. Particularly higher extraction temperatures (80°C) were used to improve the extraction efficiency in volatile phenols’ determination, a chemical family with an important role for beer aroma profile (Martins et al., 2017). However, temperatures higher than 60°C can lead to artifact formation, through Maillard reactions (Villamiel et al., 2006), thus the extraction temperature of 80°C cannot be used for the global chemical characterization of beer’s odorant molecules.

**FIGURE 5:**
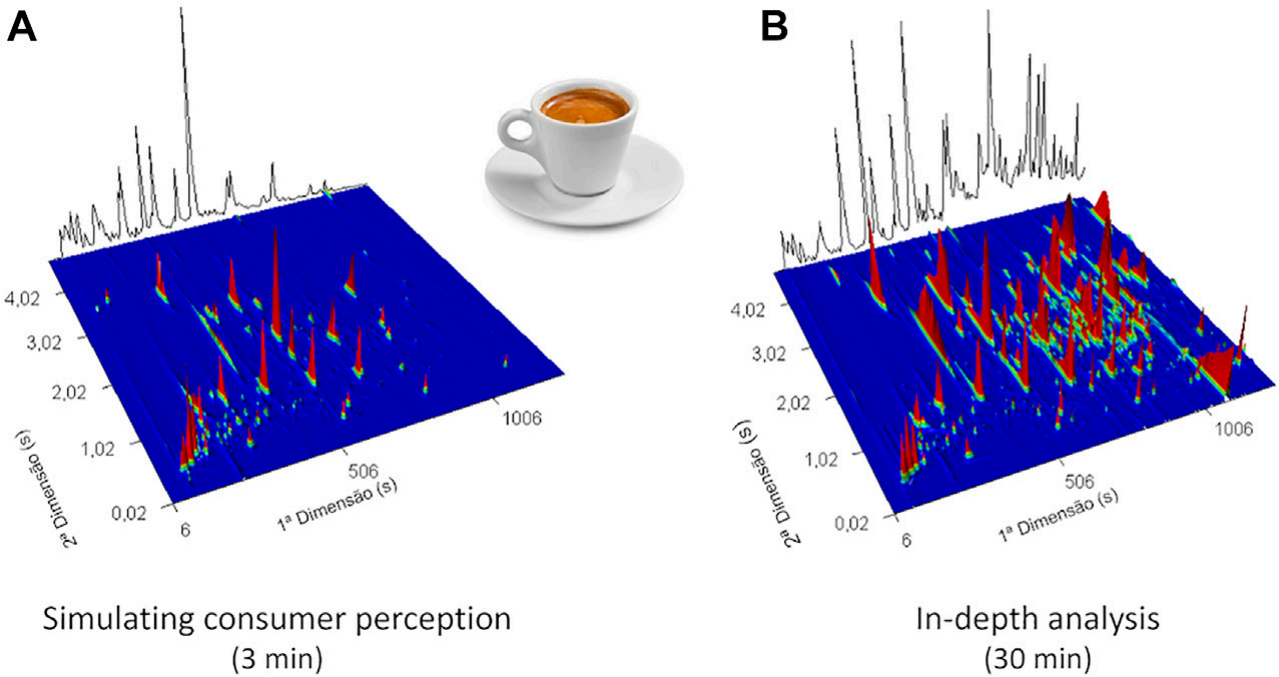
3D GC × GC total ion chromatogram plot of **(A)** espresso using 3 min of SPME extraction time to simulate the consumption conditions of this product and **(B)** with 30 min of SPME extraction time for an in-depth characterization of volatile components (using a DVB/CAR/PDMS coating fiber, at 55°C of extraction temperature).

In addition to ensuring that no artifacts are formed, it is also important to study the samples under conditions like those in which they are consumed. Indeed, the creation of the aroma clouds containing the odorant compounds is modulated among other factors by the balance between the temperature and exposure time. Therefore, selection of the optimum extraction time is one of the critical steps in the SPME method development. Extraction time selection is always a compromise between the length, sensitivity, and repeatability of the method. Equilibrium extraction provides the highest sensitivity and repeatability, but in most SPME-GC applications, pre-equilibrium conditions are used since equilibrium extraction times tend to be longer and thus impractical. Both equilibrium and pre-equilibrium extractions need precise and perfectly repeatable timing, although for the latter condition, timing is more critical (Kudlejova et al., 2009; Pawliszyn, 2009). As illustrated in Figure 5, taking advantage of the versatility of the SPME technique, different extraction times may be used, and consequently, different types of information can be extracted. For instance, 3 min of SPME extraction time allows the detection of odorants present in espresso aroma clouds and under the regular conditions of its consumption. On the other hand, longer extraction time (30 min) may be applied for an in-depth characterization of espresso volatile components (Lopes et al., 2021), from which valuable information can be extracted for blend comparison or to predict the chemical aroma profile of an espresso brew based on the respective coffee powder composition.

Salt addition and stirring effect should also be considered as important factors in the extraction efficiency, particularly in aqueous-based matrices. The mass transfer of analytes from sample matrix to headspace can be improved with salt addition through the salting-out effect, where the reduction of analytes’ solubility in the matrix occurs, while the boundary phase properties are changed (Kudlejova et al., 2009; Pawliszyn, 2009). The most used salt is high purity sodium chloride (NaCl, >99%) to avoid introduction of artifacts. First, the salting-out effect promotes the increase of extraction efficiency: hydration spheres, from water molecules, are formed around the ionic salt molecules, which leads to less free water molecules to dissolve the analytes, and consequently, analytes transfer to the headspace and then to SPME fiber coating. When saturation is achieved, the extraction efficiency may decrease because the salt ions in solution can participate in electrostatic interactions with analytes, blocking their transfer to the headspace, and consequently to SPME fiber coating (Pawliszyn, 2009). Regarding stirring effect, agitation promotes an increase of extraction efficiency through the reduction of diffusion coefficients of analytes and decreases the depletion zone effect that occurs near the fiber as a result of fluid shielding. Agitation of the sample assists the mass transfer between the sample and the fiber coating, and the time required to achieve the equilibrium can be reduced by using an agitation method (Lord and Pawliszyn, 2000; Kudlejova et al., 2009). For instance, stirring (1,000 rpm) may increase up to 3 times the chromatographic signal of compounds associated with the wine aroma when compared with analysis performed without agitation. Also, a significant increase in SPME extraction efficiency was observed, up to 6 times, which was promoted by the addition of NaCl (Rocha et al., 2001).

Despite the importance of optimizing the SPME experimental parameters to ensure a high extractive efficiency and to obtain samples’ headspace composition representativeness, innovative strategies were also developed to increase SPME performance, such as vacuum-assisted SPME and cumulative SPME. Vacuum-assisted SPME (Vac-HSSPME) consists in applying reduced pressure (usually 7 mbar, with a vacuum pump) inside a specific gas-tight sealed vial, which reduces the sampling pressure along the pre-equilibrium stage, decreasing the gas-phase resistance of analytes with low *K*_H_ values. Consequently, this procedure accelerates their extraction kinetics through the increasing of their diffusion and mass transfer to the headspace. The air-evacuation should be performed in the presence of solid matrices, while for liquid ones, the user may choose to evacuate the air before or after the introduction of the sample in the vial, which the latter option may promote the extraction of the less volatile compounds. After the sampling, SPME fiber is retracted, and then placed in the GC injector. Then, the pressure can be equilibrated to atmospheric pressure, and the vial can be opened. The main advantages of Vac-HSSPME are the requirement of shorter sampling times and lower extraction temperatures than conventional HS-SPME, thus maintaining the sample characteristics, and reducing artifact formation and compound decomposition. Otherwise, the Vac-HSSPME requires the use of a home-made gas-tight sample container (Psillakis, 2017), as no commercial options are still available. This requirement may represent a relevant constraint, as not all laboratories will be able to build this accessory. Vac-HSSPME has been applied to explore the volatiles from extra virgin olive oil (Mascrez et al., 2020), in which it was possible to provide improved information of the olive oil aroma fingerprinting (namely by obtaining higher chromatographic area), particularly due to the extraction enhancement of semi-volatile analytes by Vac-HSSPME (comparing with conventional HS-SPME). The best analytical strategy to analyze this non-aqueous liquid sample required not only Vac-HSSPME but also the use of temperature for extraction (35°C after a full factorial central composite design (CCD) analysis) to increase analytes’ diffusion through the liquid thin film (at the interface), and thus aiding their mass transfer. Another example is the use Vac-HSSPME to analyze cheese, particularly hard and semi-hard types (Sýkora et al., 2020). The main positive effects in Vac-HSSPME were achieved for the selected analytes, such as 1-pentanol, butanoic acid, 2,3-butanedione, and 2-heptanone, in water extraction; however, Vac-HSSPME only showed significant impact on butanoic acid extraction in cheese matrix. Sýkora et al. (2020) concluded that Vac-HSSPME will improve extraction of short- or medium-chain fatty acids that are present in cheese at low concentrations.

Cumulative SPME has been applied to enhance extraction performance by concentration of the volatiles through repeated sampling on the same vial, using different SPME fibers that are sequentially desorbed in the GC injector. The combination of different coatings and repeated extractions can be required once they needed to be put in the sample’s headspace at desorption time intervals, each for the established extraction time, and then they are individually desorbed. For this, the installation of a cryotrap after the injector is required to allow the volatiles’ cryofocusing (Chin et al., 2012). The main drawbacks, namely the use of different fibers, as well as the need to perform several injections, make cumulative SPME a more time-consuming and more labor-intensive technique than conventional SPME. Cumulative SPME was used to enhance SPME performance in combination with GC-O, as it was showed for wine (Chin et al., 2012, 2015) and coffee (Chin et al., 2015) characterization. Indeed, the pre-concentration of wine volatiles (e.g., retention of apolar alkanes from C_10_, and polar alcohols) improved the extract representativeness, compensating the sensitivity loss promoted by effluent splitting to two detectors (O-sensory detection, FID, or MS) (Chin et al., 2012). This effect was even more pronounced when cumulative SPME was coupled with multidimensional and comprehensive GC detection, which solved co-elutions that do not allow the proper analytes’ identification and odorant recognition (Chin et al., 2015). For instance, 2-methoxy-3-isobutylpyrazine, associated to the capsicum odor, was identified in ground coffee, and had 5-fold S/N when data were acquired using a 6-time cumulative SPME sampling and comparing with conventional SPME. Mascrez and Purcaro (2020) studied the impact of the number of cumulative SPME versus extraction time in the volatile characterization of olive oil. An improved burst level of information and overall sensitivity was achieved with shorter extraction time (10 min instead of 30 min) using 6-time cumulative SPME sampling once there was a decrease in the cumulative response using 30 min of extraction (Mascrez and Purcaro, 2020).

Even with the potential interest of these strategies, there is still a need for some improvements to allow them to be easily implemented in common food analysis laboratories, namely the development of commercial accessories (e.g., with different volumes) to perform the Vac-HSSPME. Also, cumulative SPME still requires additional investigation regarding its efficiency and sensitivity, for instance the impact of the number of cumulative sampling versus extraction time into the time of the chromatographic analysis, and consequent improvement of the sample volatile’s representativeness.

### Comprehensive Two-Dimensional Gas Chromatography

Gas chromatography is the most common technique used for food products’ and beverages’ volatile characterization, specifically the one-dimensional gas chromatography (1D-GC). Significant improvements have been occurring, driven by the need for analytical tools that can analyze target and non-target components from complex samples, from a sensitive and/or selective point of view. Thus, several advances have been performed, namely the development of new stationary phases of GC columns, improvements of chromatographic equipment (e.g., development of pneumatics, microfluidic devices, and modulators, among others) and detection systems (selective and/or high-sensitivity detectors, with increasingly compact configuration and more user-friendly maintenance), improvements in the hardware and software (Wong et al., 2013), and also the recent advances in micro-GC systems (Qu and Duan, 2019). Consequently, the improvement on resolution and limits of detection (LOD), and reduction on the time of instrumental analysis and data processing have been contributing to the deeper characterization of samples. Namely, advances on the multidimensional gas chromatography (MDGC), which was described as the process of selecting a region or zone of eluted compounds issuing from the end of one GC column, and subsequently subjecting this zone to a further GC displacement, have shown enormous advantages compared with the 1D-GC (Marriott et al., 2012; Seeley and Seeley, 2013; Prebihalo et al., 2018). MDGC are usually classified into two categories: heart-cutting 2D GC (in which only a small portion of the sample components is heart-cut and suffers both GC separations) or comprehensive two-dimensional gas chromatography (GC × GC, in which all sample components suffer heart-cutting). Both MDGC categories play an important role in aroma research of foods (Marriott et al., 2012; Seeley and Seeley, 2013; Prebihalo et al., 2018), particularly in the disclosure of aroma volatile compounds’ characterization; nevertheless, GC × GC peculiarities will be the focus of this review.

**FIGURE 6:**
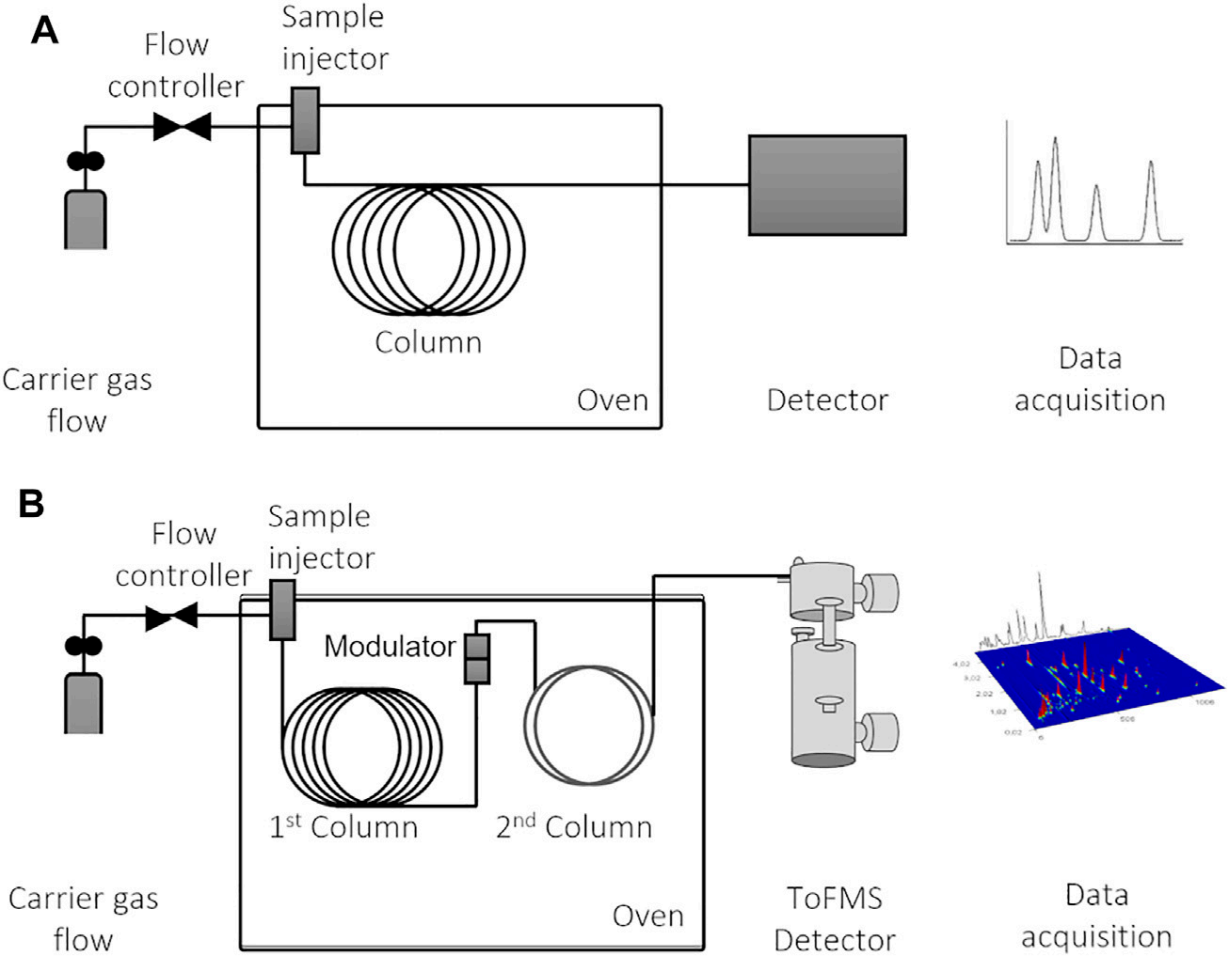
Schematic illustrations of **(A)** one-dimensional gas chromatographic system (1D-GC), which can be coupled with various types of detectors; and **(B)** comprehensive two-dimensional (GC × GC) gas chromatographic system coupled with ToFMS (time-of-flight mass spectrometry). Figure adapted from Martins et al. (2017).

This review is focused especially on the GC × GC, and for comparison purposes, Figure 6 shows a schematic representation of the two configurations of GC systems that may be used for volatile determination: 1D-GC (Figure 6A) and GC × GC-ToFMS (Figure 6B). Chromatographic systems allow the separation of mixture’s analytes through their partitioning between two phases: one large stationary surface (GC column) and a mobile phase (inert carrier gas), which is in contact with the first one. Dispersion and specific interactions between the stationary phase and the analytes are the main factors that contribute to analytes’ elution. Chromatographic column housed in a temperature-programmable oven is connected within an injector and a detector.

Despite the MS detectors commonly used and combined with GC, conventional universal, element-selective, and structure-selective detectors may also be considered. These are classified by mechanism as ionization-based (flame ionization, thermionic ionization, electron capture, photoionization, and barrier discharge), bulk physical property (thermal conductivity), optical (flame photometric, chemiluminescence, atomic emission), and electrochemical (electrolytic conductivity) detectors (Poole, 2021). MS detectors were the most widely used ones, followed by flame ionization detector (FID), and the others were generally employed for specific applications. MS detectors allow the identification and quantification of analytes within complex mixtures and promote higher sensitivity than FID. For instance, quantification of hexanoic acid in beer was performed by HS-SPME, using sol–gel-derived TMSPMA-OH-TSO and DVB/CAR/PDMS coatings, combined with GC-FID and GC-MS, respectively, and the values of 0.27 (Liu et al., 2005) and 1.1 μg/L (Rodriguez-Bencomo et al., 2012) correspond, respectively, to the LOD obtained by GC-MS and GC-FID analysis. It is important to point out that, for target analysis, very low detection and quantification limits can be achieved using specific detectors, such as for example the flame photometric detector that allowed the detection of beer sulfur compounds in the order of nanograms per liter (Xiao et al., 2006). Volatile thiols, important aroma components of the edible rapeseed oil, were successfully determined via HS-SPME (using CAR/PDMS coating) combined with GC-sulfur chemiluminescence detection, with LODs ranging from 0.16 to 1.12 μg/kg (Yang et al., 2021).

The complexity of foodstuffs and beverages usually implies long GC runs and overloaded chromatograms (with frequent co-elutions of two or more analytes), thus leading to difficulties in accurate analytes’ identification and quantification. Therefore, considerable research has been developed toward the development of independent chromatographic separations, which allow to improve the resolving power, namely GC × GC (Tranchida et al., 2004; Mondello et al., 2008; Marriott et al., 2012; Prebihalo et al., 2018). Indeed, short GC × GC runs provided more chemical information regarding the volatile profiles of foods: for instance, in the study of Brazilian chamomile (*Matricaria recutita* L.), in which a substantial reduction of the chromatographic time from 93 to only 23 min was achieved, when the analysis was performed using GC-qMS and GC × GC–ToFMS, respectively (Petronilho et al., 2011), significant improvement was observed regarding the analytes’ detection (32 instead of the 7 sesquiterpenic compounds identified by GC-qMS). Moreover, this high-throughput technique not only allowed the reduction of the chromatographic time in wine analysis but also the number of detected components: from 110 min and 51 components using GC-MS, to 44 min and 317 components using GC × GC–ToFMS (Lukić et al., 2020).

The separation efficiency of GC × GC enables an in-depth characterization of complex samples. It implies the multiple sequential separation of a sample, using two columns with different stationary phases, connected by an interface that allows to preserve the individual analytes’ retention, i.e., orthogonal mechanisms (Tranchida et al., 2004; Marriott et al., 2012) (Figure 6B). The most common GC × GC setup comprises two GC columns with different coating materials: first column (^1^D) with a length of 30 m and with a non-polar stationary phase, which separates analytes by volatility; and a second column (^2^D) short (1–2 m) and containing a polar stationary phase, which separates analytes by polarity.

This set of columns allows a relatively “slow” analytes’ separation in ^1^D column and then extremely “flash” high-resolution separation in the ^2^D column. GC columns are connected in series by a modulator. There are different types of modulators, namely thermal, cryogenic, and valve-based modulators (Bahaghighat et al., 2019). In general, the cryo-modulator, which uses liquid nitrogen for cooling, is more efficient for the analysis of the highly volatile molecules. The cryo-modulator accumulates and traps small portions (usually 4–8 s) of the eluate from the ^1^D column by cryo-focusing, and then re-injects them into the ^2^D column. The ^1^D separation is preserved once each peak is modulated several times. Moreover, the ^1^D column separation is maintained once the collected fractions are no larger than one-fourth of the peak width, thus producing narrow peaks. These peaks are consequently “flash” separated before the subsequent modulation (Tranchida et al., 2004; Marriott et al., 2012).

The reverse combination of GC columns can also provide good results, depending on the analytes to be separated. In fact, the polar/non-polar set of columns is very appropriate for the analysis of polar analytes. The column set configuration comprising ^1^D SolGel-Wax column (100% polyethylene glycol) coupled with a ^2^D OV1701 column (86% polydimethylsiloxane, 7% phenyl, 7% cyanopropyl) were utilized for the combined untargeted and targeted fingerprinting of volatiles of olive oil, and a set of saturated (heptanal, octanal, and nonanal) and unsaturated ((E)-2-heptenal) aldehydes were monitored as they were well known to be correlated with specific sensory defects of olive oils (Magagna et al., 2016). Suitable chromatographic resolution and sensitivity were achieved for a set of polar analytes (log P ranging from −1.0 to 2.8, for *N,N*-dimethylformamide and 3-octanol, respectively) using the column set configuration comprising Carbowax/BTR (ethylene glycol/siloxane copolymer) primary column coupled with Equity 5 (5% diphenyl/95% dimethyl siloxane) secondary column (Carriço et al., 2020). The use of a column set with the same diameter in primary and secondary columns yields a near-theoretical maximum in peak capacity gain, i.e., increases the number of components that the system can resolve (quantifiably and identifiably separate), and a better separation of the analytes was observed with smaller peak width.

**FIGURE 7:**
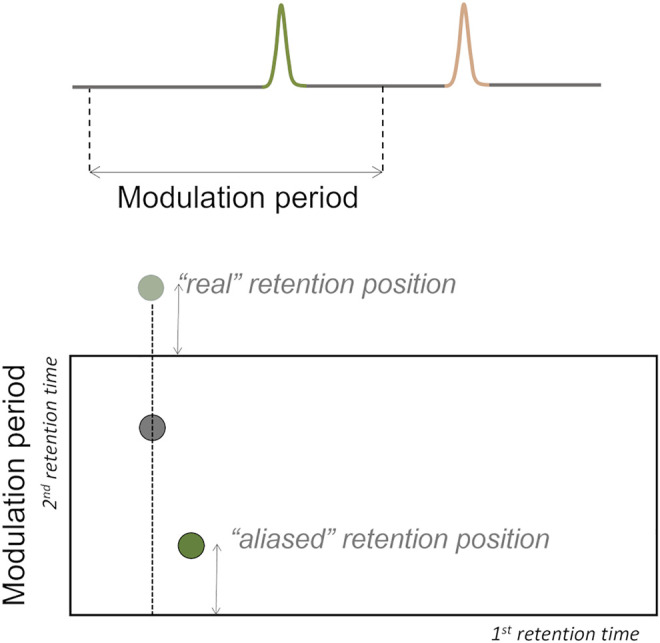
Wrap-around phenomenon may occur if the separation on the 2nd dimension does not finish before the next modulation, i.e., the elution time of the analyte exceeds the modulation time.

If the ^2^D separation of a certain analyte does not finish before the next modulation, the elution time of the analyte exceeds the modulation time, and wrap-around phenomenon occurs (Figure 7). This phenomenon can interfere in the accurate analytes’ quantification if there are co-elutions with analytes of interest (Tranchida et al., 2004; Marriott et al., 2012), thus modulation time should be previously optimized to avoid this phenomenon.

The type of detectors to be used in GC × GC depends on the requirements of rapid separation in the second dimension, i.e., the internal volume has to be small and the data acquisition rate high. For instance, the FID and electron-capture detector (ECD) have been used; nevertheless, coupling with MS detectors, especially ToF analyzers, are the most widely used and offer greater advantages in terms of compound identification (Tranchida et al., 2016, 2018; Ruiz-Matute et al., 2018). The narrow peaks produced by GC × GC (peak width at half height of 0.1 s or less) require a detector with high data acquisition speed (*ca.* hundred full-mass-range spectra per second), such as mass spectrometer, with ToF analyzer, thus providing sufficient data density (Tranchida et al., 2004; Marriott et al., 2012). Moreover, ToFMS allows the acquisition of full mass spectra at trace levels and mass spectral continuity, causing a reliable spectra deconvolution of overlapping peaks. Spectral continuity occurs when all the points of the chromatographic peak have the same ion abundance ratios for the different masses in the spectrum. More recently, progresses in the MS field go over and other MS analyzers, and more selective and sensitive ones have emerged, namely triple-quadrupole mass spectrometers, isotope ratio, and quadrupole time-of-flight mass spectrometer (Q-ToF). However, the use of low-cost GC × GC instrumentation, such as the coupling with ToFMS, would be more acceptable for the determination of the overall odorant molecules (Tranchida et al., 2016). An example of the highest sensibility of GC × GC-ToFMS can be observed, for instance in the LOD determination of eugenol, which was determined in wine matrices, with SPME (DVB/CAR/PDMS), being 0.002 and 0.01 μg/L for GC × GC-ToFMS (Welke et al., 2014) and GC-MS (Carrillo and Tena, 2006), respectively, which corresponds to a one magnitude order lower for GC × GC-ToFMS.

**FIGURE 8:**
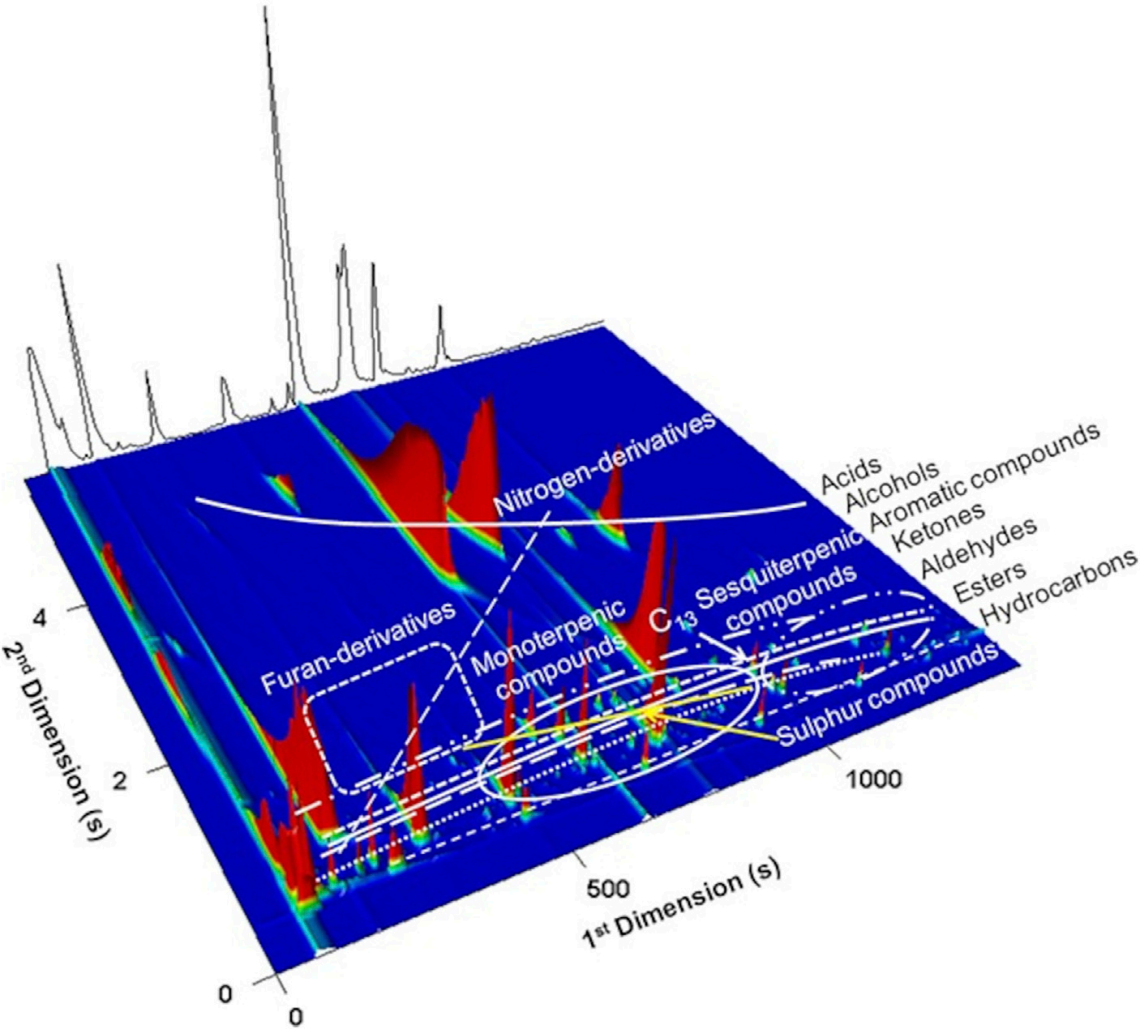
3D GC × GC total ion chromatogram plot of a Lager beer highlighting its volatile chemical families. Bands and clusters formed by structurally related compounds are indicated. Reprinted with permission from Martins et al. (2015). Copyright 2021 John Wiley and Sons, Inc.

GC × GC produces structured chromatograms due to the orthogonal analytes’ separation. For the case illustrated in Figure 8, the combination of a non-polar/polar columns configuration was used, and consequently analytes with similar chemical properties display a specific spatial location in the 2D chromatographic space. Thus, chemically related analytes are easily recognized in the 2D “chemical map,” simplifying the data processing through the reduction of analysis time and helping to obtain reliable identifications (particularly if standards are not available) (Mondello et al., 2008). These structured chromatograms are possibly one of the most important features of GC × GC, comparing with 1D-GC performance. Figure 8 elucidates the principle of the structured chromatogram, where hydrocarbons, non-polar compounds, present the lower retention time for the second dimension (^2^ *t*_R_), and acids, with higher polarity, present higher ^2^ *t*_R_ values. Due to the data complexity, specific software have been developed for GC × GC-ToFMS equipment, such as the ChromaTOF that allows the acquirement, processing, and reporting data, through the True Signal Deconvolution, and automated peak find algorithms. ChromaTOF allows to generate and to visualize the GC × GC chromatogram, either through a contour plot or a 3D plot. Figure 8 shows a 3D plot obtained from a Lager beer (Martins et al., 2015).

**FIGURE 9:**
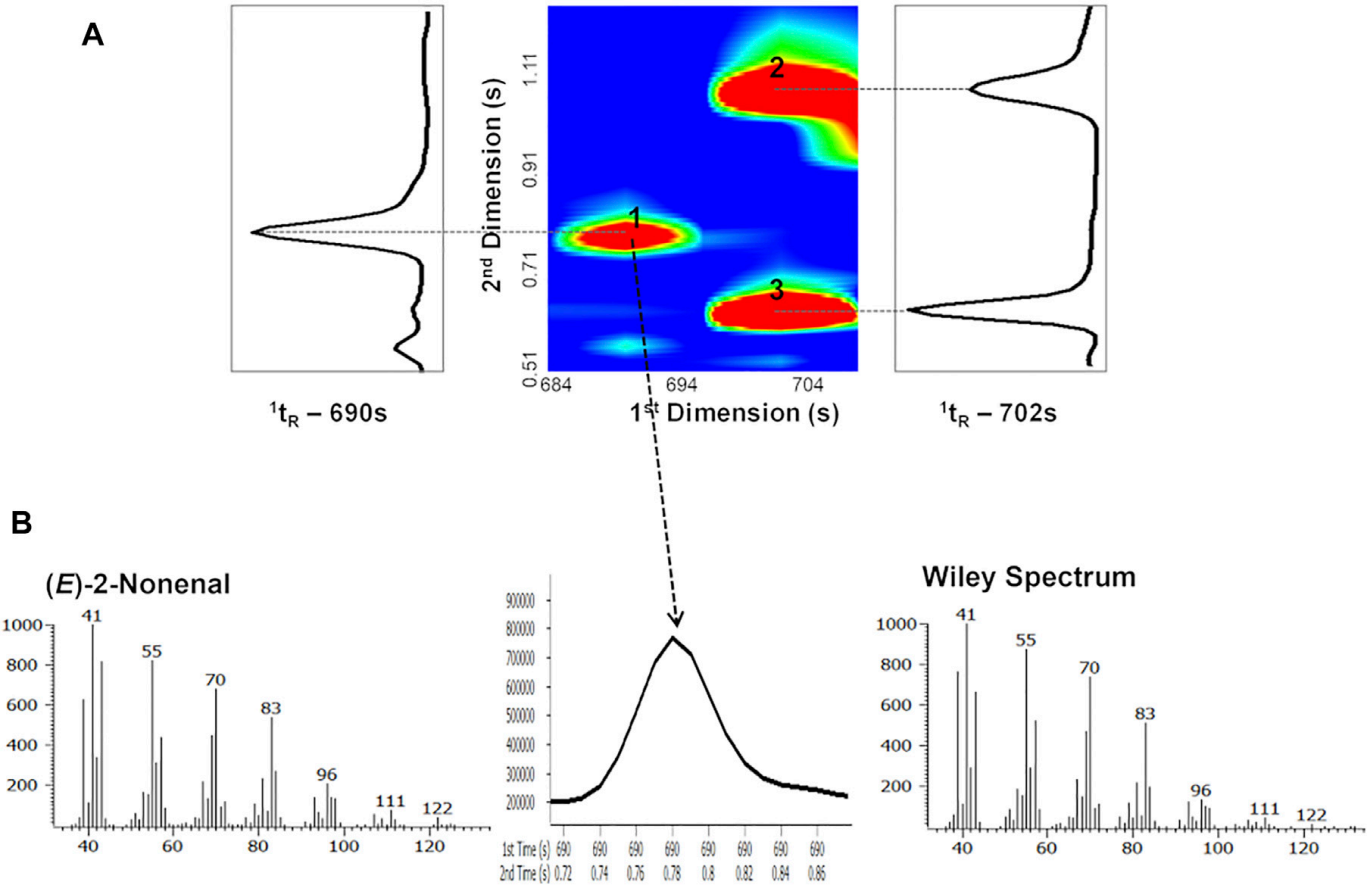
**(A)** Blow-up of a part of GC × GC chromatogram contour plot of Lager beer (obtained from Figure 8) showing the separation of 1) (*E*)-2-nonenal, 2) ethyl benzoate, and 3) 2-nonen-1-ol. **(B)** The 49-ms-wide (*E*)-2-nonenal (trace beer metabolite) GC × GC peak is easily defined and identified at a mass spectral acquisition of 125 spectra/s and its spectral quality allows its identification by comparison with mass spectrum of commercial database. Figure adapted from Martins et al. (2017).

Figure 9A represents a blow-up of a part of GC × GC total ion contour plot presented in Figure 8 and shows that compounds with similar volatility can be separated chromatographically by taking advantage of the use of a second column with a different stationary phase. Indeed, ethyl benzoate (peak 2; ^1^ *t*_R_ = 702 s, ^2^ *t*_R_ = 1.070 s) and 2-nonen-1-ol (peak 3; ^1^ *t*_R_ = 702 s, ^2^ *t*_R_ = 0.630 s) presented the same ^1^t_*R*_ (similar volatility), thus co-eluted on the Equity-5 column (^1^D). However, they exhibited different polarity and, therefore, were separated on the DB-FFAP column (^2^D), once narrow fractions were cryo-focused and re-injected on the ^2^D column, allowing the separation of two compounds within only half of a second. Comparing the chemical structures of these compounds, the differences on polarity may be explained by the presence of an aromatic ring that increases the polarity of the molecules (higher ^2^ *t*_R_), using this non-polar/polar column set.

The determination of trace analytes raises additional challenges, which may also be overcome by using GC × GC-ToFMS systems, where detection limits and spectral quality are particularly determinant. For instance, Figure 9B reveals that even with narrow peaks achieved for (*E*)-2-nonenal (*ca*. 49 ms), the spectral quality of the acquired spectrum is appropriate to compare it with MS commercial databases. Thus, the putative identification of (*E*)-2-nonenal can be achieved through the analysis of the retention times (^1^ *t*_R_ and ^2^ *t*_R_), the mass spectrum and its similarity with commercial databases, the calculation of the RI, and its comparison with the literature for column equivalents with those used in the ^1^D.

To take advantage of the potentialities of the GC × GC-ToFMS, it is important to properly define the conditions of acquisition and processing of the instrumental signal. Nowadays, robust software algorithms associated to the GC × GC-ToFMS equipment allow an easier processing of the data, regarding both qualitative and quantitative purposes. MS data are acquired using full-scan mode (i.e., data acquisition using a wide *m/z* range, where ranges from 30 to 300 *m/z* were commonly used for volatiles’ determination). In particular cases, ion extraction chromatography mode (IEC—data processing that uses data acquired in full-scan mode, and then the chromatograms may be reprocessed using specific *m/z* diagnostic ions) may be performed.

IEC may be performed by selecting a set of *m/z* diagnostic ions specific for some analytes or chemical families, allowing the definition of a two-dimensional chromatographic space containing these compounds, simplifying the data, and reducing the time of data processing. Thus, IEC contributes to increase the sensitivity and specificity, and consequently reduces the co-elution problems and increasing the GC area of specific analyte(s) (Tranchida et al., 2004). Moreover, a non-target and target analysis may be achieved with a single analysis by combining a full-scan acquisition mode with IEC data processing mode, selecting specific ions to highlight certain analyte(s) or chemical families. For instance, a HS-SPME/GC × GC-ToFMS–based methodology was developed for the global volatile analysis of Madeira wine, including age markers, with potential contribution for specific aromas of aged wines, and also allowed the quantification of ethyl carbamate (EC) (Perestrelo et al., 2010; Perestrelo et al., 2011). Under normal processing and/or storage conditions of Madeira wine, and with concentrations of urea in the range of 1–10 mg/L, a significant amount of EC may be produced. EC, known as urethane, is potentially toxic, and was re-classified in 2007 as “probably human carcinogenic compound” (Group 2A) by the International Agency for Research on Cancer, and it is recommended that its level in fortified wines should be under 100 μg/L. The reported methodology allows the EC quantification directly in Madeira wine, which provides significant reduction in time of analysis compared with the HPLC and 1D-GC methods reported in the literature (Perestrelo et al., 2010). In addition, it does not require a prior derivatization or any toxic solvent. The development of a methodology that allows the direct analysis of the sample without any previous handling is fundamental to avoid loss of analytes and the occurrence of artifacts. The use of GC × GC-ToFMS full-scan acquisition mode combined with IEC mode allowed to achieve a simultaneous quantification of EC target analyte, increasing specificity and sensitivity, and to obtain data about the furans, lactones, volatile phenols, and acetals, defined as potential aged markers (Perestrelo et al., 2010; Perestrelo et al., 2011). From these, 103 volatile compounds were tentatively identified, from which 71 have been reported for the first time in Madeira wines (Perestrelo et al., 2011). Indeed, due to the orthogonal separation, GC × GC-ToFMS system offers a more useful approach to identify these compounds compared with previous studies using GC–qMS (gas chromatography–quadrupole mass spectrometry), reducing co-elutions, increasing peak capacity and mass selectivity, and contributing to the establishment of new potential Madeira wine age markers.

The analytes’ identification must be confirmed through the co-injection of authentic standards. However, standards can be quite expensive, often unattainable in the time available for analysis, and sometimes not commercially available. Therefore, a strategy that combines several criteria is currently used to improve the identification confidence:

• Co-injection of standards (allows the construction of home-made databases),
• ^1^ *t*_R_ and ^2^ *t*_R_ values (structured chromatogram),
• Mass spectrum fragmentation pattern of each analyte and comparison with co-injected standards and MS from commercial databases (mass spectral similarity),
• Linear retention index (i.e., comparison between LRIs calculated and those available in the literature for columns like those used in the 1^st^ dimension or equivalents).

Briefly, the analytes can be tentatively identified using home-made and commercial and/or open-source mass spectra databases, such as those widely used as the Wiley 275 and US National Institute of Science and Technology (NIST). In addition, the comparison of LRI values experimentally acquired and those obtained from literature, when available, may be performed. Therefore, an *n*-alkane is given a retention index value of 100 times its carbon number. The LRI of each analyte can be computed by van Den Dool and Kratz equation (van Den Dool and Kratz, 1963), in which the analyte’s retention time is normalized with the adjacent eluting *n*-alkanes’ retention times. An *n*-alkanes series (usually ranging from C_6_ to C_20_ for volatile and semi-volatile analytes) must be injected in the same GC column and program, and the LRI values calculated for each analyte may be compared with LRIs previously reported in the literature or in open-source LRI libraries, using a GC column like the one used experimentally in the ^1^D. Indeed, different analytical laboratories can compare LRIs once they are independent of some instrumental variables of GCs (e.g., length and diameter of column, film thickness, pressure or carrier gas type, and velocity), contrary to retention times of the analytes (Babushok, 2015).

The amount of each analyte may be estimated through its chromatographic area. For target analysis, a set of methodologies may be implemented for the quantification purposes, namely construction of external calibration curves, including multiple calibration curves, and the use of isotopically labeled standards. Also, a standard addition method may be used to avoid the matrix effects. Despite providing highly accurate data, the costs associated with isotopically labeled standards have limited their use to very specific cases. On the other hand, a wide range of internal standards (IS) have been used depending on the analytes and methodologies, and on this semi-quantification approach, for each analyte, and the results are expressed as equivalent of IS. The selection of the more appropriate method for each case depends on the purpose of the work and on the characteristics of the sample. For instance, for the quantification of EC in dry/medium dry and sweet/medium sweet Madeira wines, external calibration curves were performed for both types of wines using the IEC mode (*m/z* 62). Good linearity was obtained with a regression coefficient (r^2^) higher than 0.981. Low LODs were achieved for dry (4.31 μg/L) and sweet (2.75 μg/L) wines. The quantification limits (LOQ) and recovery for dry wines were 14.38 μg/L and 88.6%, whereas for sweet wines, these were 9.16 μg/L and 99.4%, respectively. The higher performance was attained for sweet wines since the higher glucose content improved the release of volatiles to the headspace. Moreover, better linearity, recovery, and precision was achieved (Perestrelo et al., 2010).

Nowadays, some drawbacks still remain regarding the use of the GC × GC equipment, namely requiring operational expertise, complex instrumentation, and high costs of consumables (Tranchida et al., 2004; Marriott et al., 2012). However, the increase in sensitivity provided by the modulation process, together with the high chromatographic resolution power of GC × GC combined with the ToFMS, makes this technique advantageous over 1D-GC for challenging food aroma chemistry assessments. Actually, this high-throughput technique has been used to assess several data associated with aroma characteristics of foodstuffs and beverages, and multiple uses covering target and/or non-target analysis have been reported. In particular, GC × GC has been widely used in food applications, for instance for authenticity testing; search for differences between good and/or off odor, varieties or cultivars, and geographical origins; exploring for markers related with technological steps (e.g., ripening, postharvest conditions, fermentation), aroma, or aging; study of the key odorants, among others, as illustrated by the following studies: table, fortified and sparkling wines (Perestrelo et al., 2010, 2011; Weldegergis et al., 2011; Santos et al., 2013; Welke et al., 2014; Whitener et al., 2016; Rocha et al., 2021), beer (Inui et al., 2013; Martins et al., 2015, 2017, 2018), elderberry (Salvador et al., 2016), elderflower (Salvador et al., 2017), grape (Banerjee et al., 2008), aromatic plants (Shellie et al., 2002; Klimánková et al., 2008; Petronilho et al., 2011), sea salt (Silva et al., 2010, 2015), coffee (Bressanello et al., 2021; Lopes et al., 2021), hazelnut (Cordero et al., 2010; Kiefl et al., 2013; Kiefl and Schieberle, 2013; Rosso et al., 2020), honey (Čajka et al., 2007; Siegmund et al., 2018), milk (Hayward et al., 2010), cake (Rega et al., 2009), meat (Sekhon et al., 2010; Planche et al., 2015; Li W. et al., 2021), pear (Wang et al., 2019; Fonseca et al., 2020), and virgin olive oil (Peres et al., 2013; Purcaro et al., 2014; Ros et al., 2019).

### From Instrumental Data to Chemical Aroma Comprehension

**FIGURE 10:**
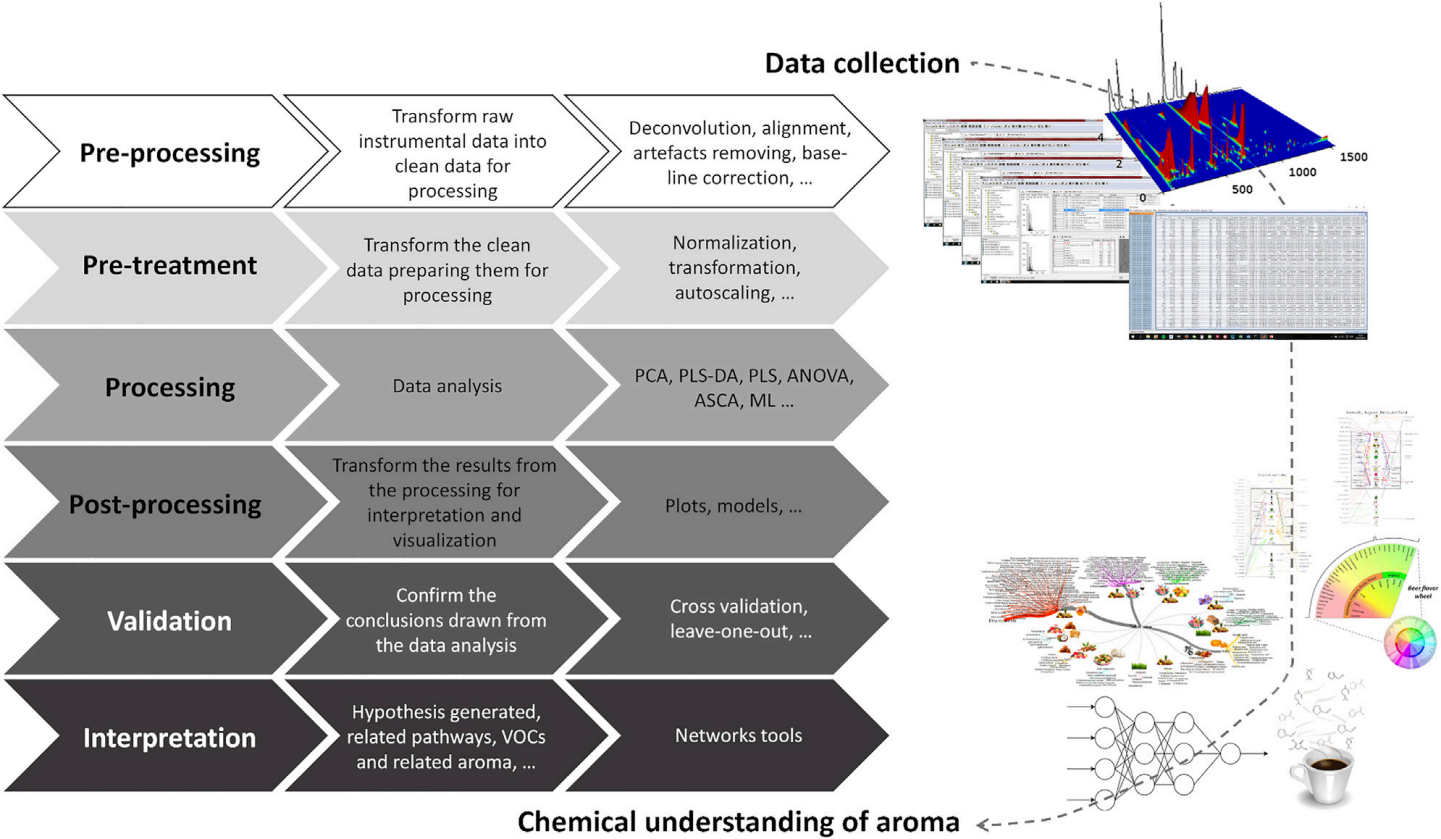
From the instrumental data collection to the chemical understanding of aroma: step-by-step data transformation. PCA—principal component analysis; PLS-DA—partial least squares-discriminant analysis; ASCA—ANOVA-simultaneous component analysis; ML—machine learning.

Considering the size and complexity of the data matrices generated by a single GC × GC-ToFMS analysis, a strategy should be carefully designed to transform the instrumental data into interpretable and useful information in understanding the aroma of food items. The main steps that may be considered on this challenging route are illustrated in Figure 10.

Data processing involves different steps from data pre-processing, pre-treatment, identification, quantification, and processing to data post-processing, validation, and interpretation. The chemical complexity of food matrices, the lack of reference mass spectra for all the compounds, and the inherent variability in each sample highlights the data processing importance. Instrumental signal data are currently used as an approach to estimate the relative content of each analyte, as it reports the response of the instrument to the analytes’ abundances. Thus, the first steps, namely the pre-processing (deconvolution, alignment, artifact removal, baseline correction, etc.) and pre-treatment (normalization, transformation, autoscaling, …), should be carefully performed before uni- and/or multivariate data analysis. Indeed, the decisions taken in these first 2 stages will have a crucial influence on the results.

Data analysis includes different possibilities, namely multivariate analysis that is intended to distinguish classes in complex datasets. Unsupervised multivariate analysis, such as principal component analysis (PCA), is often used as an exploratory technique, allowing to study the main sources of variability present in the data sets, as well as to detect clustering formation, and to establish relationships between samples (objects) and analytes (variables) (Jolliffe, 2002; Berrueta et al., 2007). Clustering methods, such as heatmap hierarchical cluster analysis, an unsupervised clustering analysis, is highly applied to evaluate the similarities among samples. By using a chromatic scale, a visual and intuitive way of displaying the content of each analyte may be achieved.

Partial least squares (PLS) is a widely used procedure for both regression and classification purposes. Concerning the classification application of PLS, known as partial least squares–discriminant analysis (PLS-DA) (Eriksson et al., 2006), the most common approach is to use a Y matrix containing dummy variables, which defines sample memberships to pre-defined groups and allows extracting relevant information/variability that could describe the reasons for the observed patterns (clusters). This methodology allows to understand which variables (analytes) contribute the most for the observed distinction (Berrueta et al., 2007). PLS-DA may be also applied combined with VIP (variable importance in projection) values to identify the main analytes that contribute for the distinction between samples. The use of VIP values allows the reduction of the data set complexity and represents a huge approach for assessing relevant information in real time. For those supervised tools, classification model complexity (number of latent variables) and classification rate and Q^2^ (quality-of-fit criterion) should be estimated by cross-validation. Thus, experiments must be carefully designed to obtain a sufficient number of samples for the construction and validation of the model(s).

ANOVA-simultaneous component analysis (ASCA) can deal with complex multivariate datasets containing an underlying experimental design. It is a direct generalization of ANOVA for univariate data to the multivariate case. The method allows for easy interpretation of the variation induced by the different factors of the design. Validation is a crucial step, especially used for supervised statistical methods (e.g., leave-one-out validation), to guarantee the reliability of the applied model. Model validation process allows demonstrating that the models obtained by the supervised pattern recognition techniques are good enough to perform classification or discrimination of the samples (Berrueta et al., 2007).

Otherwise, univariate methods are also used to study one variable or chemical family that could characterize a sample or class of samples or may be altered among them. These methods are often used as a complement of the multivariate analysis. Depending on the data set and experimental design, a wide range of methods may be used. For instance, parametric methods for data are normally distributed, as the ANOVA may be applied as a *post hoc* test, as they allow evaluating one variable for more than two conditions (samples).

Taking advantages of the bioinformatics developments, it is possible to combine different domains of information and construct networks containing several underlying factors. For instance, data analysis combining chromatographic and sensorial data may generate a visual aroma network that represents an aroma snapshot and allows to estimate the role of the different volatiles to the aroma properties of foods (Figure 10) (Ahn et al., 2011; Martins et al., 2018).

**FIGURE 11:**
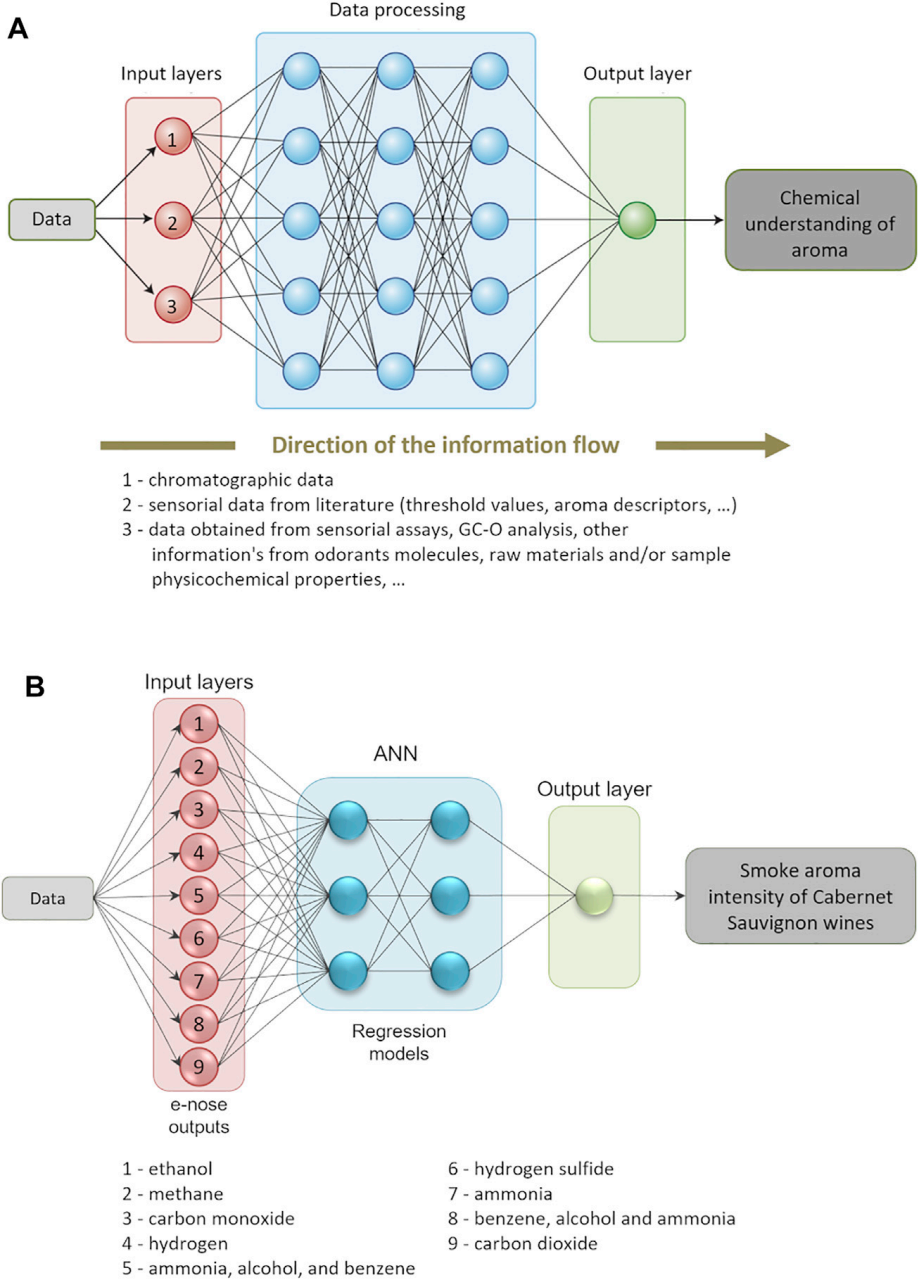
**(A)** A symbiosis between advanced instrumental and bioinformatics with sensorial analysis or other data will represent a stream advance in chemical understanding of food aromas, taking advantage of the machine learning (ML) principles. Figure adapted from Topol (2019). **(B)** Assessment of volatile compounds in smoke-tainted Cabernet Sauvignon wines using a low-cost e-nose and machine learning modeling. The high accuracy regression models were constructed using e-nose outputs as inputs to predict smoke aroma intensity of Cabernet Sauvignon wines. ANN—artificial neural network. Figure adapted from Summerson et al. (2021a).

More recently, innovative advances have been applied for data analysis, namely machine learning (ML), which is a subset of the artificial intelligence techniques, and that utilizes algorithms to extract patterns in a diversity of applications (Telikani et al., 2022). In this context, ML can be applied to unraveling the food aroma using the complex datasets resultant from the comprehensive fingerprinting and/or profiling of foods (for instance, as those obtained by GC × GC). ML techniques can use different domains as input (called units, nodes, or neurons), such as chromatographic data, sensorial data from literature (e.g., odor thresholds and aroma descriptors, among others), and/or data resultant from sensorial analysis, GC-O, and e-nose, among others. The pattern analysis is then applied to those inputs, using different algorithms (uses the weight of the link between two units), that can lead to the chemical understanding of aroma (Figure 11A). Several parameters should be optimized to achieve an accurate prediction model without over-fitting (i.e., good fitting results for the training dataset in the learning phase, however untrusty results with testing data), for instance the number of samples, hidden layers, and the number of iterations in the network’s learning phase, among others (Hategan et al., 2021). If the dataset requires improvement of robustness (e.g., low number of samples), the data augmentation technique can be applied, for instance, to perform the convolutional neural network (CNN) based on deep chemometrics (Bjerrum et al., 2017).

ML has been applied to unveil the specific aroma attributes of different food matrices, such as wine and beer. Various ML algorithms [namely linear discriminant analysis (LDA), linear support vector machines (SVM), and quadratic SVM] were applied to the comprehensive volatile fingerprinting extracted by GC×GC-ToFMS from 127 wines (Reichenbach et al., 2019). Those ML methods were able to classify wines according to grape varieties, grape-growing regions, vintage years, and wineries. For example, β-eudesmol presented higher Fisher ratio for a specific winery, and its presence in the wines may be linked to the closeness of the vineyards to *Eucalyptus* forests (this volatile compound is characteristic of *Eucalyptus* trees), whose leaves may take part accidentally in vinification. Furthermore, a rapid, non-destructive, and cost-effective tool was developed using 2 ML models based on artificial neural networks (ANNs) (Summerson et al., 2021a). The high accuracy regression models were constructed using e-nose outputs as inputs to predict the level of the volatile compounds and the smoke aroma intensity of Cabernet Sauvignon wines, thus creating a decision-making tool for winemakers (Figure 11B). The same approach was also used to access the effectiveness of smoke taint amelioration treatments in Pinot Grigio wines (Summerson et al., 2021b), and the more effective treatment was through the cleaving enzyme coupled with activated carbon, which can help delay the recovery of smoke aromas in wine’s subsequent aging and glycoconjugate hydrolysis.

A highly accurate ML model (R = 0.98) based on ANN was developed to predict consumers’ beer acceptability (Viejo et al., 2019). This model used the relative peak area of volatile data obtained as input; the aroma, flavor, and overall liking from sensorial analysis were used as targets from 24 analyzed beers. The ANN Bayesian regularization algorithm demonstrated to be a more appropriate analysis than multiple regression models once it included nonlinear correlations between the inputs and targets previously enumerated. The developed model allowed, for instance, to observe that 4-ethyguaiacol and *trans*-β-ionone contributed positively to beer, regarding aroma, flavor, and overall liking, while styrene contributed to the opposite effect. ML models can be also integrated with other technologies such as sensors and robotics, as it was demonstrated by the rapid and accurate evaluation of commercial beers using low-cost robotics and sensors—RoboBEER (Viejo and Fuentes, 2020). For this case, the absorbance of near-infrared (NIR) spectroscopy was used as input to predict the peak areas of volatiles and chemical parameters of beers; also, the same approach was used using robotics (RoboBEER) as input. Both achieved models were accurate; nevertheless, they still require improvement, such as complement the ML model with other robust data, including GC-MS, GC-O, and/or e-nose data.

Other pioneering approaches have attracted the attention of the food aroma specialists in recent years, namely the sensomics that is a reference procedure to characterize the key food odorants (KFOs), which includes the identification and accurate quantitation of KFOs, aroma reconstitution, and omission experiments (Schieberle and Hofmann, 2011). Different methodologies can be used for this purpose, such as solvent-assisted flavor evaporation (SAFE), to extract the food’ volatiles; aroma extract dilution analysis (AEDA) to assign the aroma quality and volatiles’ potency, through the preparation of different diluted extracts to be further analyzed by GC-O; and stable isotope dilution assays to quantify the volatile components with highest aroma potency, and consequently their OAV can be calculated. Model matrices are then spiked with the pure compounds of the identified KFOs (using the determined concentrations in the food itself) to prepare the aroma recombinates. These aroma recombinates are sensorially evaluated and compared with the original food, by a trained sensory panel, which validate the KFOs, considering their accurate identification and quantitation (Schieberle and Hofmann, 2011; Nicolotti et al., 2019). Thus, KFOs are the food volatile components that, on one hand, exceed their OT in the food matrix and, on the other hand, have a determinant role to mimic the real food sensory profile, after the recombination and omission experiments.

Sensomics has been used to determine KFOs in a wide range of foods, for instance, black tea (Schieberle and Hofmann, 2011), cognac (Uselmann and Schieberle, 2015), truffles (Schmidberger and Schieberle, 2017), yeasted wheat dough (yeast dumpling) (Sahin and Schieberle, 2019), pretzels’ crust (Schoenauer and Schieberle, 2019), wine (Lyu et al., 2019), fried bread (Lasekan and Dabaj, 2020), garlic (Abe et al., 2020), bagels (Lasekan et al., 2021), fermented soybean product (Zhao et al., 2021), or cheese (Wang et al., 2021). However, some drawbacks can be pointed out, which makes this approach hard to be used as routine analysis, namely it is time consuming and globally complex, and it requires a wide range of different analytical instruments and techniques (Schieberle and Hofmann, 2011; Nicolotti et al., 2019).

Based on the sensomics approach, a single analytical platform was developed called sensomics-based expert system (SEBES) that foresees KFOs in an extract, through the combination of odor thresholds and quantification data in one software that automatically calculate the OAVs. This alternative and fast approach does not presuppose the use of human olfactory system, and the food odor codes are achieved by artificial intelligence smelling (Nicolotti et al., 2019), which gives a good alternative for routine analysis. Moreover, GC × GC-ToFMS and GC Image software are recommended for SEBES approach: the former due to its high resolution and high sensitivity; the latter because of the software’s capacity to perform automated quantitative data based on features and other custom functions (e.g., database construction of odor thresholds of KFOs in several matrices). SEBES was successfully applied to rum and Cabernet Sauvignon wines, once its performance was compared with the conventional sensomics approach (Nicolotti et al., 2019) and good agreement was observed between the two approaches (differences below 20%). Some instrumental drawbacks of SEBES are still required to overcome, e.g., shift to headspace over liquid techniques to detect highly volatile compounds; some odor thresholds are inferior to instrument’s sensitivity and are not quantified as KFOs (Nicolotti et al., 2019). More recently, a headspace-based technique (MHS-SPME-GC × GC-MS/FID) was proposed as artificial intelligence smelling machine using extra virgin oil as the studied matrix (Stilo et al., 2021), thus overcoming the drawback presented by Nicolotti et al. (2019). Despite the already respectable level of software automatization, there is still a great margin for future improvement, for instance developments presupposing the use of ML, which can lead to machine decision-making. Also, the construction of open access odor threshold databases will pursue the better development of SEBES approach (Nicolotti et al., 2019).

## Concluding Remarks and Future Perspectives

This review provides the state-of-the-art and the technical know-how for young researchers and an extensive range of specialists from the food-related area and others who want to start studying aroma of food items at chemical level based on the use of advanced gas chromatographic methodologies. Indeed, increase in knowledge of aroma chemistry science was powered by advances in chemical analysis, being the gas chromatography the central technique to characterize odorant compounds. Also, the vapor above a food—aroma cloud—holds the odorant molecules that may be perceived by the odor receptor sites of the smell organ. SPME is a sample preparation technique well suited to the extraction of these molecules.

As a capstone, it was considered important to highlight and/or systematize the main ideas addressed in this review:

• SPME, a solvent-free extraction technique, allows in a single step the sampling, extraction, and concentration of headspace volatile components, i.e., the aroma clouds components. Currently, there are several stationary phases and geometries on the market, and new solutions are under development. 3D printing will undoubtedly be one of the solutions that can open new opportunities in extracting representative headspace components,
• SPME/GC × GC–based methodologies overcome a set of challenges previously reported for the analysis of volatile and semi-volatile food items; namely, it allows the detection of a high number of chemical structures of volatile and semi-volatile molecules, in a wide concentration range (from pg to mg),
• GC × GC-ToFMS combined with SPME seems to be an appropriate combination to unveil food aroma complexity, as the modulation process (i.e., the cryofocusing of analytes between the two GC columns) and sensitivity achieved from ToFMS will compensate the non-exhaustive extraction provided by SPME,
• GC × GC–based methodologies allow a simultaneous targeted and detailed study of the aroma clouds volatile composition within a single analysis, contributing to the detection of a huge number of analytes as never before achieved,
• This throughput issue is convenient for the analysis of large numbers of samples in a very convenient time (*ca*. 20 min of instrumental acquisition per sample),
• The generated instrumental raw data are compatible with the advanced data analysis tools, and some of them are free to access. Advanced artificial intelligence techniques may be used to interpret the volatile signatures and classify them based on data from collected smells, namely by combined chemical and sensorial data,
• The innovative strategies presented to transform the chromatographic data into useful information for aroma comprehension, although not replacing the sensorial assays, generate very important information for the understanding of the aroma at the level of the molecule and the phenomena related to its origin and/or open new approaches to modulate, modify, or even create new aromas.

Despite the enormous advantages covered by GC × GC comparing with 1D-GC, such as improved resolution and peak capacity, faster run times, and improved detection limits, its use is still restricted to very specific applications. The new generations of GC × GC are designed to reduce some drawbacks, namely making maintenance operations easier for the user, smaller equipment, more compact and easily integrated in common laboratories, and advances in modulators to reduce the costs of consumables, among others. Due to the size and complexity of the data generated in a single analysis by GC × GC, the data processing steps are time-consuming and tedious and there are some limitations that must be overcome to make this technique more appealing for the users. Also, to go further on the aroma chemistry comprehension, the combination of chromatographic data with other domains of information is crucial. Thus, the following are identified as very pertinent:

• Development of tools for the instrumental signal alignment, and in a relatively quick way to identify and quantify high numbers of analytes, in a large number of samples,
• Creation of open access databases with information about aroma potential of volatile and semi-volatile molecules (i.e., aroma descriptor and threshold value) in a wide range of food products, particularly for endogenous and unique products whose knowledge and valuation can have a high impact on the society and economy of local communities,
• Development of a bioinformatics platform that in an integrated way can allow the acquisition of instrumental signals and their processing, including interaction with databases of sensory information (when applicable). Indeed, in the future and after training, these tools will ingest and meaningfully process massive sets of data quickly, accurately, and inexpensively, and for machines that will see and do things that are not humanly possible.

For a holistic understanding of the aroma of foods, it is crucial to define a broad strategy, involving diverse techniques that can assess the multiple dimensions of the aroma perception, that are intrinsically associated with the multimodal perception concept, i.e., multimodal phenomena concern stimuli that generate simultaneous (or nearly simultaneous) information in more than one sensory modality (Chambers IV and Koppel, 2013; Bressanello et al., 2021). Certainly, the chemical aroma data are not enough, but crucial to move forward on this challenging topic, and GC × GC-ToFMS seems to be a powerful technique for the analytical coverage of the chemical clouds of food. This technique seems to fulfill the requirements of the innovative strategies in the field of the aroma chemistry such as the smell digitalization. Due to the huge significance of human olfaction in several fields, namely to improve nutritional health, diagnose and treat diseases, understand consumer preferences and consumption, measure and chemically reveal the smells, it represents cutting-edge research with an increase of such a tendency to be expected in the future.

Aroma chemistry is under an ever-increasing knowledge on chemical data driven by the latest equipment and bioinformation innovation. Although this review has been focused on the aroma of food, the concepts, methodologies, and challenges discussed in this context are perfectly transposable to the study of a wide range of non-food items. This information at the molecular level can be exploited in several dimensions beyond aroma, as these data may be useful to solve or understand issues related with product and process control, food safety, establishment of botanical markers, and understanding of the individual’s response in health and disease condition, or in specific age range, among others.
